# Associations of birth weight and later life lifestyle factors with risk of cardiovascular disease in the USA: A prospective cohort study

**DOI:** 10.1016/j.eclinm.2022.101570

**Published:** 2022-07-18

**Authors:** Yi-Xin Wang, Yanping Li, Janet W. Rich-Edwards, Andrea A. Florio, Zhilei Shan, Siwen Wang, JoAnn E. Manson, Kenneth J. Mukamal, Eric B. Rimm, Jorge E. Chavarro

**Affiliations:** a Department of Nutrition, Harvard T.H. Chan School of Public Health, Boston, MA, USA; b Department of Epidemiology, Harvard T.H. Chan School of Public Health, Boston, MA, USA; c Division of Women's Health, Department of Medicine, Brigham and Women's Hospital, Harvard Medical School, Boston, MA, USA; d Department of Nutrition and Food Hygiene, Hubei Key Laboratory of Food Nutrition and Safety, School of Public Health, Tongji Medical College, Huazhong University of Science and Technology, Wuhan, China; e Channing Division of Network Medicine, Department of Medicine, Brigham and Women's Hospital and Harvard Medical School, Boston, MA, USA; f Division of Preventive Medicine, Department of Medicine, Brigham and Women's Hospital and Harvard Medical School, Boston, MA, USA; g Department of Medicine, Beth Israel Deaconess Medical Centre, Harvard Medical School, Boston, MA, USA

**Keywords:** Cardiovascular disease, Birth weight, Lifestyle

## Abstract

**Background:**

Low birth weight has been associated with a greater risk of cardiovascular disease (CVD). However, the interaction between low birth weight and adult lifestyle factors on the risk of CVD remains unclear.

**Methods:**

We included 20,169 men from the Health Professionals Follow-up Study (HPFS, 1986–2016), 52,380 women from the Nurses’ Health Study (NHS, 1980–2018), and 85,350 women from the Nurses’ Health Study II (NHS II, 1991–2017) in the USA who reported birth weight and updated data on adult body weight, smoking status, physical activity, and diet every 2–4 years. Incident cases of CVD, defined as a combined endpoint of fatal and nonfatal coronary heart disease (CHD) and stroke, were self-reported and confirmed by physicians through reviewing medical records.

**Findings:**

During 4,370,051 person-years of follow-up, 16,244 incident CVD cases were documented, including 12,126 CHD and 4118 stroke cases. Cox proportional hazards regression models revealed an increased risk of CHD during adulthood across categories of decreasing birth weight in all cohorts (all *P* for linear trend <0.001). Additionally, we found an additive interaction between decreasing birth weight and unhealthy lifestyles on the risk of CHD among women, with a pooled relative excess risk due to interaction of 0.06 (95% CI: 0.04–0.08). The attributable proportions of the joint effect were 23.0% (95% CI: 11.0–36.0%) for decreasing birth weight alone, 67.0% (95% CI: 58.0–75.0%) for unhealthy lifestyle alone, and 11.0% (95% CI: 5.0–17.0%) for their additive interaction. Lower birth weight was associated with a greater stroke risk only among women, which was independent of later-life lifestyle factors.

**Interpretation:**

Lower birth weight may interact synergistically with unhealthy lifestyle factors in adulthood to further increase the risk of CHD among women.

**Funding:**

The National Institutes of Health grants.

**Research in context**

**Evidence before this study**

Low birth weight, a proxy measure of fetal intrauterine growth restriction, has been associated with a greater risk of cardiovascular disease (CVD). We performed a systematic search in PubMed and Web of Science from inception until March 22, 2022, using search terms (“birth weight”) AND (“interaction” OR “body mass index” OR “tobacco use” OR “nutrition” OR “physical activity”). However, very few studies have explored the potential interaction between birth weight and adult lifestyle factors on the subsequent risk of CVD, particularly on an additive scale.

**Added value of this study**

Results from three large prospective cohorts consistently revealed that birth weight was inversely associated with the risk of developing coronary heart disease in later life, particularly among women who adopted an overall unhealthy lifestyle during adulthood. The association of birth weight with stroke risk was less consistent, which persisted only among women and was independent of later-life lifestyle factors.

**Implications of all the available evidence**

Our findings support the Developmental Origins of Health and Disease hypothesis suggesting the potential long-term health consequences of an adverse intrauterine environment and point to potential lifestyle interventions to reduce the risk of developing CVD among individuals with low birth weight.

Alt-text: Unlabelled box

## Introduction

Cardiovascular disease (CVD), including coronary heart disease (CHD) and stroke, killed an estimated 18.6 million people in 2019.^1^ The annual global economic burden of CVD is estimated to increase by 16% from $906 billion in 2015 to more than $1 trillion in 2030.^2^ In addition to traditional risk factors occurring during adolescence and adulthood, increasing evidence suggests that fetal and infant life could be critical periods for the development of CVD later in life.^3^ Low birth weight (LBW; less than 2500 g), a proxy measure of fetal intrauterine growth restriction, has been associated with CVD morbidity and mortality, based on a large body of evidence from cross-sectional surveys, case-control studies, registry databases, prospective cohorts, and Mendelian randomization analyses.^4^, ^5^, ^6^, ^7^, ^8^, ^9^, ^10^, ^11^, ^12^, ^13^, ^14^, ^15^, ^16^, ^17^

Extensive evidence from randomized clinical trials and high-quality cohort studies shows that lifestyle factors including physical activity, tobacco use, nutrition, and overweight or obesity are important determinants of CVD.^18^ Therefore, the 2019 American College of Cardiology/American Heart Association Guideline on the Primary Prevention of Cardiovascular Disease has incorporated these lifestyle factors into the recommendations to prevent CVD.^19^ We have previously reported that among women participating in the Nurses’ Health Study (NHS), the inverse association between birth weight and CHD was stronger among participants who had higher body mass index (BMI) in adulthood.^4^ Similarly, several studies also reported an association between birth weight and CHD among men restricted to those with high BMI in adulthood.^10^^,^^11^ To date, however, very few studies have explored the potential interaction between low birth weight and BMI, tobacco use, nutrition, and physical activity throughout adulthood on the subsequent risk of CVD, particularly on an additive scale that is important for identifying biologic mechanisms (e.g., synergism or antagonism between two exposures) and improving preventive interventions.^20^ Therefore, we assessed the interaction between birth weight and the overall lifestyle factors in adulthood on the risk of incident CVD both on multiplicative and additive scales among men from the Health Professionals Follow-up Study (HPFS) and women from NHS and the Nurses’ Health Study II (NHS II).

## Methods

### Study population

The HPFS (*n* = 51,529), NHS (*n* = 121,700), and NHSII (*n* = 116,429) are ongoing prospective cohort studies established in 1986, 1976, and 1989 in the USA, respectively, by recruiting male and female health professionals. Participants are followed biennially via postal or electronic questionnaires that collect data on demographic characteristics, lifestyle factors, and incident diseases. The follow-up response rate of each cycle exceeds 90% in all three cohorts. The initial food frequency questionnaire was completed by participants in HPFS, NHS, and NHS II in 1986, 1980, and 1991, respectively, which, served as the analysis baseline. The study protocol was approved by the institutional review boards of the Brigham and Women's Hospital and the Harvard TH Chan School of Public Health (Protocol number: 2009-P-002375). Returning a completed questionnaire indicates informed consent.

**Figure 1:**
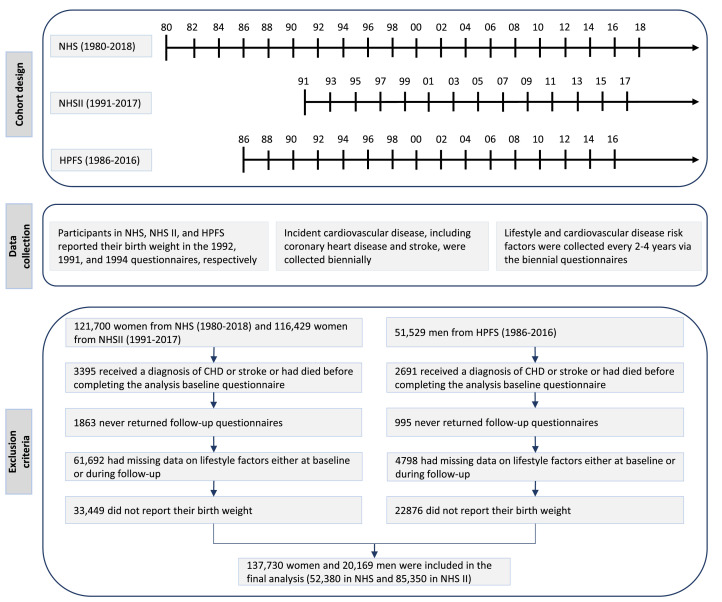
**Flow diagram for cohort design, data collection, and exclusion criteria**. Abbreviations: HPFS= the Health Professionals Follow-up Study (*n* = 20,169); NHS=the Nurses’ Health Study (*n* = 52,380); NHS II=the Nurses’ Health Study II (*n* = 85,350).

We excluded participants who received a diagnosis of CHD or stroke at baseline to avoid retrospective collecting CVD events that may lead to reverse causation and non-differential recall bias. We also excluded those who had died or did not provide information about their birth weight at analysis baseline, or had missing data on smoking, BMI, diet, alcohol consumption, or physical activity either at baseline or during follow-up, leaving 20,169 men (12.77%) and 137,730 women (87.23%) for the current analysis (Figure 1). Baseline characteristics and crude incidence of CVD were similar between included participants and those excluded due to a lack of data on birth weight (Table S1).

### Assessment of birth weight

Participants in NHS reported their birth weight as “less than 5.0 pounds (lbs)”, “5.0 to 5.5 lbs”, “5.6 to 7.0 lbs”, “7.1 to 8.5 lbs”, “8.6 to 10.0 lbs”, “more than10.0 lbs”, and “not sure” on the 1992 questionnaire. Similarly, in the NHS II and HPFS cohorts, birth weight was reported as “less than 5.5 lbs”, “5.6 to 7.0 lbs”, “7.1 to 8.5 lbs”, “8.6 to 10.0 lbs”, “more than 10.0 lbs”, and “unknown” in the 1991 and 1994 questionnaires, respectively. Thus, the birth weight categories in our current analysis were (in kg): <2.5, 2.5-3.15, 3.16-3.82, 3.83-4.5, and >4.5. Women in NHS and NHS II also reported whether they were multiple births (e.g., twins and triplets) or born two or more weeks premature (gestational period less than 38 completed weeks). Among 3803 participants from HPFS, 68.6% reported the same birthweight category as reported by their mothers.^21^ The mean birth weight reported by HPFS participants and their mothers was very similar (7.65±1.25 vs. 7.63±1.17 lb), with a Spearman correlation coefficient of 0.71 (*P* < 0.001).^21^ Similarly, among 220 randomly selected women from NHS II, 70.0% reported the same birthweight category as was obtained by state birth records.^21^ The Spearman correlations of self-reported birthweight with recalled birthweight by their mothers (*n* = 528) and state birth records (*n* = 220) were 0.75 and 0.74, respectively.^21^

### CVD Ascertainment

CVD was defined as a combined endpoint of fatal and nonfatal CHD, including nonfatal myocardial infarction and coronary revascularization (coronary bypass or angioplasty), and stroke.^22^ Fatal CHD or stroke was defined as CHD or stroke listed as the cause of death on the death certificate, which has been demonstrated to ascertain more than 98% of deaths. We used the International Classification of Diseases (ICD), eighth and ninth revision, to identify deaths due to cardiovascular disease, including coronary heart disease, stroke, heart failure, and any other vascular causes (ICD-8 codes 390-458; ICD-9 codes 390-459).^23^ Nonfatal incident events and diagnosis dates were reported on each biennial questionnaire, which was confirmed by physicians through reviewing medical records with participants’ consent. Nonfatal myocardial infarction was identified according to the World Health Organization criteria.^22^ Nonfatal stroke was identified according to the National Survey of Stroke criteria.^22^ The diagnosis of coronary artery bypass graft surgery was self-reported, which has been demonstrated to ascertain more than 96% of physician-adjudicated cases in men from the HPFS cohort.^24^

### Assessment of lifestyle factors and covariates

Information on height and race/ethnicity were self-reported at baseline. Body weight, marital status, and health conditions (e.g., menopausal status for women, family history of CVD, and living status) were self-reported at recruitment and updated every 2 or 4 years thereafter. We calculated BMI for each follow-up cycle. Maternal and paternal smoking status while living together with them during childhood and maternal history of diabetes and hypertension were self-reported via the biennial questionnaires. Women in NHS and NHSII also reported their maternal and paternal occupation and homeownership at the time of the nurse's birth. Physical activity was reported approximately every 2 years in men and 4 years in women. We calculated the total hours per week spent in moderate to vigorous activities (including brisk walking) that require the expenditure of ≥3 metabolic equivalents of task per hour.^25^ Dietary intake, including alcohol intake, was self-reported every 4 years using a validated semiquantitative food frequency questionnaire. Given that the Dietary Approaches to Stop Hypertension (DASH) diet was recommended as an effective nutritional strategy to prevent CVD,^26^ we created the DASH score as a summary measure of the overall diet quality. We classified participants into high-risk groups according to their time-varying BMI (≥25 kg/m^2^), smoking status (current smokers), diet quality (in the bottom 60% of DASH diet score), physical activity (<30 minutes/day of moderate-to-vigorous intensity activity), and alcohol consumption (non-moderate).^21^ To keep consistency with our previous studies,^21^^,^^25^ moderate alcohol consumption was defined as 5–30 g/day for men and 5-15 g/day for women according to the 2015–2020 alcohol intake guideline for Americans. For each time-varying lifestyle factor, each participant received a score of 1 if they met the criterion for high risk; or 0 otherwise. We added each lifestyle score to generate the time-varying overall unhealthy score. The reliability and validity of self-reported weight, levels of physical activity, diet intake, and smoking status have been identified to be highly reliable among participants in HPFS, NHS, or NHSII.^21^^,^^25^

### Statistical analysis

Since there was no violation of the proportional hazard assumption based on the likelihood ratio test by adding an interaction term between birth weight and follow-up time, we used time-dependent Cox proportional hazards regression models to estimate the hazard ratios (HRs) and 95% confidential intervals (CI) for the associations of birth weight categories (<2.5, 2.5-3.15, 3.16-3.82, 3.83-4.5, and >4.5 kg) with the risk of CVD (see Supplemental Appendix). Participants contributed follow-up periods from the date of returning the analysis baseline questionnaire until the date of diagnosis of CVD, death, or end of follow-up (January 2016 in HPFS, June 2018 in NHS, and June 2017 in NHS II), whichever occurred first. To control for age, calendar time, and any possible interactions between these two timescales, all analyses were stratified jointly by age in months at the start of follow-up and the calendar year for the current questionnaire cycle.^23^ Multivariable Cox models were adjusted for race/ethnicity, family history of CVD, as well as time-varying marital and living status, menopausal status and postmenopausal hormone therapy use (women only), alcohol consumption, BMI, physical activity, smoking status, and DASH diet score. The Anderson-Gill data structure was applied to efficiently handle time-varying covariates by creating new data records for each follow-up cycle at which participants were at risk.^23^ Participants in the middle category of birth weight (3.16–3.82 kg) were treated as the reference group. Tests for linear trends were conducted by modeling birthweight categories as an ordinal variable by assigning the median value to each category. The potential non-linear association between birth weight and risk of CVD was also assessed using restricted cubic splines.^21^

To evaluate whether the joint effect of lower birth weight and unhealthy lifestyle together was larger than the product of individual effects, we assessed the multiplicative interaction by adding a cross-product term between any tested factors and birth weight in the multivariable Cox models. We also assess whether the joint effect of two factors together was larger than the sum of the individual effects of lower birth weight and unhealthy lifestyles, we estimated additive interaction by calculating the relative excess risk due to interaction (RERI) using the SAS codes developed by Li and Chambless.^27^ RERI was calculated as (HR11−HR10−HR01)+1, where HR11 is the HR of CVD associated with having both exposures, HR10 is the HR of CVD associated with decreasing birth weight alone, and HR01 is the HR of CVD associated with unhealthy lifestyles alone.^27^ The confidence interval for RERI was estimated for statistical inferences by using the standard delta method.^27^ We also decomposed the joint effect by estimating the proportions attributable to birth weight alone, unhealthy lifestyle alone, and their interaction.^27^ We performed these above-mentioned analyses separately in each cohort to improve confounding control and then pooled the hazard ratios to obtain summarized risk estimations using an inverse variance weighted, random effect meta-analysis and applied the Cochran's Q statistic and the I^2^ statistic to test the heterogeneity of estimations between cohorts.

Several sensitivity analyses were conducted. First, we reanalysed the association between birth weight and CVD risk by classifying the participants who were multiple births (e.g., twins and triplets) or born two or more weeks premature into a separate exposure category to assess if the associations were independent of multiple pregnancies or preterm birth. Second, we additionally included maternal and paternal occupation and homeownership at the time of the nurse's birth in multivariable Cox models to assess the potential influence of early-life socioeconomic status. We restricted these above-mentioned analyses to women in NHS and NHS II because data on multiple births, preterm birth, and parental occupation and homeownership was not collected among men from HPFS. Third, we included participants from HPFS, NHS, and NHS II who had missing data on lifestyle factors by creating a missing indicator in the multivariable Cox models to examine whether the exclusion could have biased the results. Fourth, we additionally included adult height, maternal and paternal smoking status, and maternal history of diabetes and hypertension in multivariable Cox models. Fifth, we excluded BMI from the calculation of overall unhealthy score to assess the combined influence of all other lifestyles. Sixth, we recalculated the overall unhealthy score by classifying participants who consumed alcohol less than 5 g/day into low-risk groups. All data were analysed using SAS 9.4 for UNIX (SAS Institute Inc).

### Role of the funding source

The funders had no role in the design and conduct of the study; collection, management, analysis, and interpretation of the data; preparation, review, or approval of the manuscript; or the decision to submit the manuscript for publication. Y-XW, LP, and JEC have access to the dataset and all authors have final responsibility for the decision to submit for publication.

## Results

Our study included 20,169 men in HPFS, 52,380 women in NHS, and 85,350 women in NHS II, whose mean (SD) age, at analysis baseline, was 51.69 (9.02), 45.40 (7.12), and 36.00 (4.66) years, respectively. In total, 999 (5.0%) participants in HPFS, 5577 (10.7%) in NHS, and 6707 (7.9%) in NHS II reported a birth weight below 2.5 kg (Table 1). Within each cohort, the lowest baseline BMI was observed among participants in the second category of birth weight (2.5-3.15 kg). The proportion of current smokers increased across the categories of increasing birth weight in the HPFS and NHS cohorts, whereas decreasing in the NHS II cohort.

**Table 1:** Baseline characteristics of study participants according to birthweight category.

Characteristics	Birthweight category (kg)				
	<2.5	2.5-3.15	3.16-3.82	3.83-4.5	>4.5
The Health Professionals Follow-up Study (1986)					
Number of participants	999	4591	9526	3567	1486
Age (y)	52 (9.4)	51.2 (8.8)	51.2 (8.9)	51.9 (9.1)	55.5 (9.1)
Height (m)	1.8 (0.1)	1.8 (0.1)	1.8 (0.1)	1.8 (0.1)	1.8 (0.1)
BMI (kg/m^2^)	25.4 (3.4)	25.2 (3.2)	25.4 (3.1)	25.9 (3.3)	26.2 (3.2)
Total energy intake, kcal/d	1967.9 (591.5)	1992.5 (620.8)	2027.2 (620.4)	2051.3 (634.4)	2042.1 (623.5)
DASH diet score	23.8 (5.5)	24.1 (5.5)	24.2 (5.4)	24.1 (5.3)	24.3 (5.5)
Alcohol intake, g/d	10.7 (14.9)	11 (14.7)	11.7 (15.3)	11.8 (15.3)	11.3 (15.3)
Current smoking, %	8.7	7.7	8.6	8.9	10.0
Moderate to vigorous intensity exercise, h/wk	2.6 (3.5)	2.9 (4.1)	3.1 (5)	2.9 (3.9)	3.1 (5.2)
Currently married, %	90.8	90.5	91.5	90.5	92.0
The Nurses’ Health Study (1980)					
Number of participants	5577	16225	23610	5769	1199
Age (y)	45.1 (7.1)	45.2 (7.1)	45.3 (7.1)	46.3 (7.2)	48.3 (6.7)
Height (m)	1.6 (0.1)	1.6 (0.1)	1.6 (0.1)	1.7 (0.1)	1.7 (0.1)
BMI (kg/m^2^)	24.3 (4.6)	23.9 (4.2)	24.4 (4.4)	24.8 (4.7)	25.4 (5)
Premenopausal, %^a^	58.9	58.8	59.7	58.3	55.9
Total energy intake, kcal/d	1546.9 (496.9)	1566.2 (492.7)	1575.9 (491)	1563.1 (501.3)	1554.2 (514.3)
DASH diet score	23.9 (4.6)	23.8 (4.6)	23.9 (4.6)	24 (4.6)	23.9 (4.5)
Alcohol intake, g/d	6 (10.1)	6.4 (10.3)	6.5 (10.5)	6.3 (10.3)	6.2 (10.4)
Current smoking, %	26.9	26.8	26.8	28.4	29.8
Moderate to vigorous intensity exercise, h/wk	4 (2.9)	4 (2.9)	4.1 (2.9)	4 (2.9)	3.9 (2.8)
Currently married, %	72.7	73.6	73.2	72.9	74.7
The Nurses’ Health Study II (1991)					
Number of participants	6707	25919	41284	10381	1059
Age (y)	36.7 (4.6)	36 (4.7)	36 (4.7)	35.6 (4.7)	36.2 (4.5)
Height (m)	1.6 (0.1)	1.6 (0.1)	1.7 (0.1)	1.7 (0.1)	1.7 (0.1)
BMI (kg/m^2^)	24.6 (5.5)	24.4 (5.2)	24.6 (5.2)	25.1 (5.5)	25.9 (6.2)
Premenopausal, % a	95.7	96.6	96.6	96.5	97.0
Total energy intake, kcal/d	1791.8 (562.1)	1778.4 (547.7)	1798.8 (544.5)	1802.9 (543.5)	1801.1 (557.6)
DASH diet score	23.5 (5)	23.5 (5)	23.6 (4.9)	23.7 (5)	23.9 (5.1)
Alcohol intake, g/d	3 (6.2)	3.1 (6.2)	3.2 (6)	3 (5.9)	2.8 (5.4)
Current smoking, %	13.3	12.4	12.1	11.8	10.3
Moderate to vigorous intensity exercise, h/wk	2.4 (3.7)	2.4 (3.8)	2.4 (3.8)	2.4 (3.6)	2.6 (4.5)
Currently married, %	77.0	77.7	79.4	77.7	75.7

Values are means (SD) or percentages. All variables except age are age-standardized.

a Women who reported that their menstruation had ceased as a result of surgery, radiotherapy, or chemotherapy were not categorized in the premenopausal group. Abbreviations: DASH=Dietary Approaches to Stop Hypertension.

**Figure 2:**
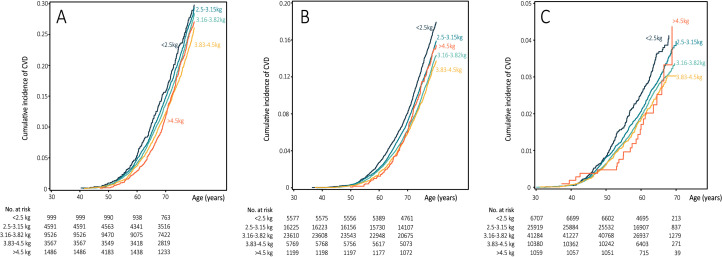
**Crude cumulative incidence of cardiovascular disease according to birthweight category**. A: the Health Professionals Follow-up Study (*n* = 20,169); B: the Nurses’ Health Study (*n* = 52,380); and C: the Nurses’ Health Study II (*n* = 85,350).

**Figure 3:**
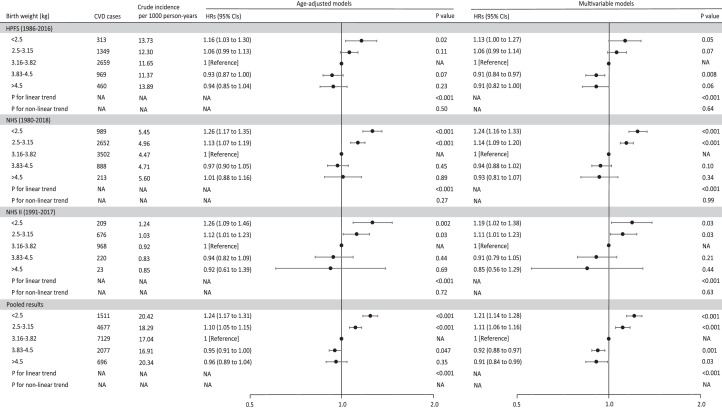
**Hazard ratio (95% CI) of cardiovascular disease according to birth weight among men in the Health Professionals Follow-up Study (***n* = **20,169) and women in the Nurses’ Health Study (***n* = **52,380) and the Nurses’ Health Study II (***n* = **85,350)**. In the age-adjusted model, age in months (continuous) at the start of follow-up and the calendar year of the current questionnaire cycle were included as stratified variables to control for potential confounding by age, calendar time, and any possible interactions between these two timescales. Multivariable models were further adjusted for ethnicity (white, yes/no), family history of CVD (yes/no), as well as time-varying marital status (yes/no), living status (alone or not), menopausal status [premenopausal or postmenopausal (never, past, or current menopausal hormone use), women only], smoking status (never smoker, former smoker, current smoker: 1-14, 15-24, ≥25 cigarettes/d), alcohol drinking (0, 0.1-4.9, 5.0-14.9, 15.0-19.9, 20.0-29.9, ≥30 g/d), exercise (0, 0.01-1.0, 1.01-3.49, 3.5-5.99, ≥6 h/week), DASH diet score (5 categories), and body mass index (<21, 21-24.9, 25-29.9, 30-31.9, ≥32 kg/m^2^). *P*-values for the between-study test of heterogeneity were all above 0.05. P for nonlinearity was tested using restricted cubic splines. Abbreviations: NA=not applicable; HR= Hazard ratio; CI = confidence interval; HPFS= the Health Professionals Follow-up Study; NHS=the Nurses’ Health Study; NHS II=the Nurses’ Health Study II.

During 4,370,051 person-years of follow-up, 16,244 incident CVD cases were documented in all three cohorts, including 12,126 CHD and 4,118 stroke cases. The crude cumulative incidence of CVD was the highest among participants whose birth weight was below 2.5 kg in all cohorts (Figure 2). Similarly, the Cox models showed an increased risk of CVD during adulthood across the categories of decreased birth weight (all *P* for linear trend <0.001; Figure 3). In the final multivariable models with adjustment for confounding and well-established risk factors, participants with a birth weight of <2.5, 2.5-3.15, 3.83-4.5, and >4.5 had pooled multivariable HRs for CVD during follow-up of 1.21 (95% CI: 1.14 to 1.28), 1.11 (95% CI: 1.06 to 1.16), 0.92 (95% CI: 0.88 to 0.97), and 0.91 (95% CI: 0.84 to 0.99), respectively, compared with participants in the middle category of birth weight (3.16-3.82 kg). When CHD and stroke were separately evaluated (Table S2 and Table 2), we observed a consistently inverse association between birth weight and CHD risk both in men and women (all P for linear trend <0.001). Compared with participants in the middle category of birth weight (3.16-3.82 kg), participants with birth weight of <2.5, 2.5-3.15, 3.83-4.5, and >4.5 kg had pooled multivariable HRs for CHD during follow-up of 1.25 (95% CI: 1.17 to 1.33), 1.12 (95% CI: 1.06 to 1.19), 0.91 (95% CI: 0.86 to 0.97), and 0.91 (95% CI: 0.83 to 1.00), respectively (Table 2). Low birth weight was also associated with higher stroke risk among women in NHS and NHS II (Table S2), which was not observed among men (P for heterogeneity by sex=0.12; Table 2).

**Table 2:** Hazard ratio (95% CI) of CHD and stroke according to birth weight among men in the Health Professionals Follow-up Study (*n* = 20,169) and women in the Nurses’ Health Study (*n* = 52,380) and the Nurses’ Health Study II (*n* = 85,350).

	Birthweight category (kg)					P for linear trend
	<2.5	2.5-3.15	3.16-3.82	3.83-4.5	>4.5	
CHD						
**Men**						
Cases	283	1191	2307	841	394	-
Crude incidence, per 1000 person years	12.40	10.85	10.10	9.86	11.88	-
HR (95% CI) in age-adjusted models *	1.21 (1.07 to 1.37)	1.07 (1.00 to 1.15)	1 [Reference]	0.94 (0.87 to 1.01)	0.95 (0.85 to 1.05)	<0.001
HR (95% CI) in multivariable models †	1.17 (1.04 to 1.33)	1.08 (1.01 to 1.16)	1 [Reference]	0.91 (0.84 to 0.98)	0.91 (0.82 to 1.01)	<0.001
**Women**						
Cases	852	2307	3045	744	162	-
Crude incidence, per 1000 person years	2.37	2.08	1.84	1.87	2.20	-
HR (95% CI) in age-adjusted models *	1.31 (1.21 to 1.41)	1.14 (1.08 to 1.20)	1 [Reference]	0.95 (0.88 to 1.03)	1.01 (0.86 to 1.19)	<0.001
HR (95% CI) in multivariable models †	1.27 (1.18 to 1.37)	1.16 (1.09 to 1.22)	1 [Reference]	0.92 (0.85 to 1.00)	0.92 (0.78 to 1.07)	<0.001
**Pooled results based on meta-analyses**						
HR (95% CI) in age-adjusted models *	1.28 (1.20 to 1.36)	1.11 (1.06 to 1.17)	1 [Reference]	0.94 (0.89 to 1.00)	0.97 (0.88 to 1.06)	<0.001
P for heterogeneity‡	0.61	0.31	-	0.94	0.50	0.20
HR (95% CI) in multivariable models †	1.25 (1.17 to 1.33)	1.12 (1.06 to 1.19)	1 [Reference]	0.91 (0.86 to 0.97)	0.91 (0.83 to 1.00)	<0.001
P for heterogeneity‡	0.46	0.21	-	0.97	0.62	0.22
Stroke						
**Men**						
Cases	30	174	374	131	68	-
Crude incidence, per 1000 person years	1.30	1.57	1.63	1.53	2.04	-
HR (95% CI) in age-adjusted models *	0.76 (0.52 to 1.10)	0.97 (0.81 to 1.16)	1 [Reference]	0.88 (0.72 to 1.07)	0.90 (0.69 to 1.16)	0.84
HR (95% CI) in multivariable models †	0.76 (0.52 to 1.10)	0.99 (0.82 to 1.18)	1 [Reference]	0.85 (0.70 to 1.04)	0.88 (0.67 to 1.14)	0.59
**Women**						
Cases	358	1056	1477	370	80	-
Crude incidence, per 1000 person years	1.00	0.95	0.89	0.93	1.10	-
HR (95% CI) in age-adjusted models *	1.16 (1.03 to 1.30)	1.08 (1.00 to 1.17)	1 [Reference]	0.97 (0.87 to 1.09)	1.02 (0.81 to 1.28)	0.004
HR (95% CI) in multivariable models †	1.14 (1.01 to 1.28)	1.09 (1.01 to 1.18)	1 [Reference]	0.95 (0.85 to 1.07)	0.98 (0.78 to 1.23)	0.002
**Pooled results based on meta-analyses**						
HR (95% CI) in age-adjusted models *	1.06 (0.86 to 1.31)	1.07 (0.99 to 1.15)	1 [Reference]	0.95 (0.86 to 1.05)	0.96 (0.81 to 1.14)	0.008
P for heterogeneity‡	0.11	0.40	-	0.63	0.70	0.43
HR (95% CI) in multivariable models †	1.05 (0.86 to 1.28)	1.07 (1.00 to 1.15)	1 [Reference]	0.93 (0.84 to 1.02)	0.93 (0.79 to 1.11)	0.003
P for heterogeneity‡	0.12	0.49	-	0.60	0.75	0.58

⁎ In age-adjusted models, age in months (continuous) at the start of follow-up and calendar year of the current questionnaire cycle were included as stratified variables to control for potential confounding by age, calendar time, and any possible interactions between these two timescales.

† Models were further adjusted for ethnicity (white, yes/no), family history of CVD (yes/no), as well as time-varying marital status (yes/no), living status (alone or not), menopausal status [premenopausal or postmenopausal (never, past, or current menopausal hormone use), women only], smoking status (never smoker, former smoker, current smoker: 1-14, 15-24, ≥25 cigarettes/d), alcohol drinking (0, 0.1-4.9, 5.0-14.9, 15.0-19.9, 20.0-29.9, ≥30 g/d), exercise (0, 0.01-1.0, 1.01-3.49, 3.5-5.99, ≥6 h/week), DASH diet score (5 categories), and body mass index (<21, 21-24.9, 25-29.9, 30-31.9, ≥32 kg/m^2^).

‡ Test for between-study heterogeneity. Abbreviations: CHD=coronary heart disease; CI=confidence interval.

Multiplicative interaction was only observed between birth weight and BMI on the risk of CVD among men in HPFS (Table S3). Nevertheless, we observed a positive additive interaction between decreasing birth weight and the overall unhealthy score on the risk of CHD among women, but not in men (Table S4 and Table 3). Similarly, the increased risk of CVD associated with lower birth weight appeared to be stronger among participants reporting a greater number of unhealthy lifestyle factors in women when we jointly categorized participants according to birth weight and the overall lifestyle score (Figure S1). The pooled relative excess risk of CHD due to the interaction between per kg lower birth weight and the overall unhealthy lifestyle score was 0.06 (95% CI: 0.04 to 0.08) in women (Table 3). The attributable proportions of the joint effect were 23.0% (95% CI: 11.0% to 36.0%) for decreasing birth weight alone, 67.0% (95% CI: 58.0% to 75.0%) for unhealthy lifestyles alone, and 11.0% (95% CI: 5.0% to 17.0%) for their additive interaction (Table 3). When each individual lifestyle factor was separately assessed, the additive interaction persisted only between decreasing birth weight and overweight or obesity, smoking, and non-moderate alcohol intake among women (Tables S5 and Table S6).

**Table 3:** Attributing effects to additive interaction between birth weight and lifestyles on risks of CHD and stroke among men in the Health Professionals Follow-up Study (*n* = 20,169) and women in the Nurses’ Health Study (*n* = 52,380) and the Nurses’ Health Study II (*n* = 85,350).*

	Men	Women	Pooled results	P for heterogeneity^‡^
CHD				
Main effect				
Lower birth weight (per kg)	1.14 (1.05 to 1.24)	1.13 (1.02 to 1.25)	1.13 (1.07 to 1.21)	0.99
Time-varing unhealthy lifestyle score (1-4)†	1.23 (1.16 to 1.31)	1.39 (1.23 to 1.58)	1.32 (1.20 to 1.45)	0.04
Joint effect	1.37 (1.27 to 1.47)	1.58 (1.41 to 1.78)	1.50 (1.34 to 1.68)	0.03
Measures of interaction				
Relative excess risk due to interaction	0.004 (-0.03 to 0.04)	0.06 (0.04 to 0.08)	0.05 (0.01 to 0.09)	0.02
P for additive interaction	0.83	<0.001	0.04	0.07
P for multiplicative interaction	0.35	0.64	0.75	0.61
Attributable proportion, %				
Lower birth weight	37.0% (24.1 to 49.9%)	23.0% (11.0 to 36.0%)	29.0% (19.0 to 40.0%)	0.27
Unhealthy lifestyles	62.0% (53.4 to 70.6%)	67.0% (58.0 to 75.0%)	65.0% (60.0 to 70.0%)	0.32
Additive interaction	1.0% (-8.8 to 10.9%)	11.0% (5.0 to 17.0%)	8.0% (2.0 to 15.0%)	0.23
Stroke				
Main effect				
Lower birth weight (per kg)	1.07 (0.87 to 1.31)	1.10 (0.96 to 1.25)	1.09 (0.97 to 1.22)	0.83
Time-varing unhealthy lifestyle score (1-4) †	1.22 (1.05 to 1.42)	1.26 (1.14 to 1.39)	1.25 (1.15 to 1.36)	0.64
Joint effect	1.27 (1.03 to 1.51)	1.41 (1.19 to 1.66)	1.35 (1.21 to 1.51)	0.34
Measures of interaction				
Relative excess risk due to interaction	-0.02 (-0.12 to 0.08)	0.02 (-0.02 to 0.06)	0.01 (-0.02 to 0.05)	0.48
P for additive interaction	0.67	0.67	0.45	0.44
P for multiplicative interaction	0.60	0.88	0.70	0.85
Attributable proportion, %				
Lower birth weight	25.5% (-29.0 to 80.0%)	28.0% (6.0 to 50.0%)	28.0% (7.0 to 48.0%)	0.92
Unhealthy lifestyles	82.5% (54.5 to 110.5%)	68.0% (58.0 to 79.0%)	70.0% (60.0 to 80.0%)	0.52
Additive interaction	-8.0% (-38.9 to 22.6%)	5.0% (-7.0 to 17.0%)	3.0% (-8.0 to 14.0%)	0.74

⁎ Cox proportional hazards models were adjusted for age, ethnicity (white, yes/no), family history of CVD (yes/no), as well as time-varying marital status (yes/no), living status (alone or not), menopausal status [premenopausal or postmenopausal (never, past, or current menopausal hormone use), women only].

† Unhealthy lifestyles include currently smoking, exercising <30 min/d at moderate intensity, DASH diet score in the bottom three fifths, body mass index ≥25 kg/m^2^, and not moderate alcohol consumption (moderate: 5-15 g alcohol/d in women, 5-30 g alcohol/d in men). ^‡^Test for between-study heterogeneity; for each lifestyle factor, each participant received a score of 1 if they met the criterion for high risk which were summarized to calculate the overall unhealthy score. Abbreviations: CHD=coronary heart disease; CI = confidence interval.

The association between birth weight and CVD risk persisted when we included participants who had missing data on lifestyle factors (Table S7), when we additionally included adult height, maternal and paternal smoking status, maternal history of diabetes and hypertension, participants’ socioeconomic status during infancy as covariates in the multivariable models (Tables S8–S10), and when we classified participants who were multiple births (e.g., twins and triplets) or born two or more weeks premature into a separate exposure category (Table S11 and S12). The additive interaction of decreasing birth weight and the overall unhealthy score with the risk of CHD among women persisted when we excluded BMI from the calculation of the overall unhealthy lifestyle score and when we classified participants who consumed alcohol less than 5 g/day into low-risk groups (Table S13 and S14).

## Discussion

Results from three large prospective cohorts all revealed that birth weight was inversely associated with subsequent risk of CHD during adulthood, which supports the Developmental Origins of Health and Disease (DOHaD) hypothesis showing that the earliest stages of human development — even during intrauterine life— could be critical periods for the development of chronic diseases later in life.^3^ More importantly, we observed an additive interaction between decreasing birth weight and overweight and obesity, smoking, and non-moderate alcohol intake on CHD risk among women. The association of birth weight with stroke risk, however, was less consistent, which persisted only among women and was independent of later-life lifestyle factors.

While some studies have not reported an association between birth weight and CHD risk,^28^ our findings are consistent with the preponderance of evidence from cross-sectional surveys,^6^ case-control studies,^8^ registry databases,^11^^,^^12^^,^^16^ prospective cohorts,^4^^,^^5^^,^^9^^,^^10^^,^^13^, ^14^, ^15^ and Mendelian randomization analyses,^17^ showing an inverse association in both men and women. In support of our findings, Wang and colleagues did not find any evidence of sex differences in the inverse association between birth weight and CHD in a previous meta-analysis based on 27 prospective cohort studies.^29^ The association of birth weight with stroke risk, however, was less consistent. In our present study, birth weight was inversely associated with stroke risk among women, which was in agreement with earlier reports from this cohort and other female populations.^4^^,^^5^ Very few studies to date have explored the association between birth weight and subsequent risk of stroke among men. In contrast with two small studies based on hospital register databases that reported an inverse association between birth weight and male stroke risk,^30^^,^^31^ birth weight was unrelated to stroke among men from HPFS. The inconsistent results between studies might be partly related to the differences in population characteristics and sample size. For instance, compared to women in NHS, participants in HPFS were fewer and had a lower prevalence of incident stroke. As a result, only 777 men in HPFS (2612 in NHS) received a diagnosis of stroke during follow-up, which may have been insufficient to generate precise estimations. Besides, previous register studies did not collect detailed data on various relevant confounders and lifestyle factors during adulthood that might have resulted in unmeasured confounding or uncontrolled modifying effect.

While extensive evidence has revealed that lifestyle factors, including tobacco use, nutrition, physical activity, and overweight or obesity, are important for preserving good cardiovascular health over the life course,^19^ very few studies to date have explored the interaction between birth weight and these lifestyles on the risk of CVD. Our study based on three large cohorts revealed that the inverse association between birth weight and CVD was not totally dependent on later-life lifestyle factors, given that stratified analyses showed similar results among participants who were physically active, maintained a lean body shape, never smoked, ate a high-quality diet, and drank moderate levels of alcohol. However, we found positive interaction on the additive scale between decreasing birth weight and unhealthy lifestyles on the risk of CHD among women (NHS/NHS II), suggesting that the association of low birth weight with the increased risk of CHD is greater among women who have adopted an overall unhealthy pattern of lifestyles. The excess risk of CHD due to lower birth weight and overweight or obesity, smoking, and non-moderate alcohol intake was higher than the summed risk associated with low birth weight and each individual lifestyle factor, suggesting that fetal growth restriction might interact synergistically with adult unhealthy lifestyles to further increase the risk of CHD. In support of our finding, Li and colleagues reported additive interaction between an unfavorable lifestyle profile and low birth weight on cardiometabolic diseases among 19,779 twins from the Swedish Twin Registry.^32^ There was no evidence of additive interaction between birth weight and lifestyle factors on the risk of CHD among men in HPFS. This sex-specific association warrants further research but might be related to the sex difference in the effect of prenatal and adulthood risk factors on CHD. In our earlier study of an overlapping population, the additive interaction between birth weight and unhealthy lifestyles in relation to type 2 diabetes was stronger among women in NHS/NHS II than men in HPFS.^21^ Interestingly, multiplicative interaction showed that the inverse association of birth weight with CVD was only observed among men whose BMI was less than 25 kg/m^2^. Overweight and obesity have been strongly associated with a greater risk of CVD,^33^ which may have obscured the association between birth weight and CVD. However, given that very few HPFS participants had a birth weight less than 2.5 kg [2.64% (533 of 20,169) for BMI≥25 kg/m^2^; 2.31% (466 of 20,169) for BMI<25 kg/m^2^], chance findings cannot be fully ruled out.

The observed inverse associations of birth weight with subsequent CVD risk in adulthood may reflect shared mechanistic pathways in utero where metabolic stress leads to insulin resistance,^34^ decreased leptin levels,^35^ and altered intracellular insulin signaling pathways.^36^ These programmed alterations in function have been associated with the development of cardiometabolic risk (e.g., obesity, hypertension, and insulin resistance) in adulthood,^36^ which in turn might be responsible for the disruptions to cardiovascular systems in later life. In addition, low birth weight may be served as a marker of aberrant intrauterine environment related to prenatal malnutrition, maternal cigarette use, pregnancy complications, and genes. For instance, exposure to specific nutrient deficiency during pregnancy has been associated with reduced numbers of nephrons,^37^ altered glucocorticoid activity,^38^ and disrupted hypothalamic-pituitary-adrenal (HPA) axis activity,^1^ which are important pathogeneses of cardiovascular and metabolic disorders. The fetus may also respond to the aberrant intrauterine environment through some metabolic and vascular adaptations, such as insulin resistance, endothelial dysfunction, increased allocation of energy to the development of vital organs (e.g., brain and heart), and reduced skeletal mass and bone mineralization, which would result in lifelong changes in the cardiovascular system.^3^^,^^7^ The interaction of fetal growth restriction and overweight or obesity, smoking, and low-quality diet with the risk of CHD is also biologically plausible. Women with lower birth weight are more likely to have an adverse cardiovascular risk or metabolic profile,^21^ which may have been further exacerbated by later life unhealthy lifestyles.

Strengths of this study include the replication of the results across three cohorts, prospective design with long-term follow-up periods, a large number of participants and incident medical record-validated CVD, and the collection of various potential confounders (e.g., preterm birth) and lifestyle factors. The association between birth weight and CVD risk persisted when we excluded participants who were multiple births or born two or more weeks premature, indicating that low birth weight is an independent risk factor for CVD. Our study also has some limitations. First, our validation analysis showed that there is some misclassification of self-reported birth weight. In this case, however, such misclassification is likely to be non-differential with respect to incident CVD because of the prospective design of all cohorts, resulting in risk estimations biased towards the null. Second, a large proportion of participants were excluded due to missing data on birth weight, which may have induced selection bias. However, similar baseline characteristics and crude incidence of CVD were observed between included participants and those excluded due to missing data on birth weight. Compared to participants included in our current analyses, the crude and adjusted pooled HR of CVD among excluded participants was 1.02 (95% CI: 0.88, 1.17) and 1.02 (95% CI: 0.93, 1.11), respectively. Third, despite the adjustment for multiple potential confounders, residual and unmeasured confounding cannot be fully ruled out. Fourth, our cohort participants were all health professionals and had a relatively homogeneous racial/ethnic and educational attainment, which may limit the generalizability of our findings. Fifth, we did not consider the presence of competing risks,^39^ although our data set contained censored observations who had not yet developed CVD up to the censoring time. These censored observations, however, should be very limited given the inclusion of fatal CHD and stroke as primary endpoints and the high follow-up rate of each biennial cycle in three cohorts (all >90%). Finally, despite strong accumulating evidence showing that intrauterine growth is associated with a greater risk of CVD, our observational studies cannot demonstrate causality. However, as it is impossible to randomize participants to different birth weight categories and lifestyle intervention groups, long-term prospective cohort studies with high quality such as ours will be the best available data for exploring the health consequences of birth weight and adult lifestyle factors.

In summary, results from three large prospective cohorts consistently showed an inverse association between birth weight and the risk of developing CHD in later life, particularly among women who adopted an overall unhealthy lifestyle. We also find evidence that lower birth weight may interact synergistically with unhealthy lifestyles in adulthood to further increase the risk of CHD. The inverse association between birth weight and stroke persisted only among women and was independent of lifestyles. Our results emphasize the potential long-term health consequences of an adverse intrauterine environment and point to potential lifestyle interventions to reduce the risk of developing CVD among women with low birth weight.

## Contributors

Y.-X.W. analyzed and drafted the manuscript. Y.-X.W. and J.E.C. were involved in the study conception and design. Y.L. verified the underlying data and conducted a technical review. JEC obtained funding for the study. Y.-X.W., Y.L., J.W.R.-E., A.A.F., Z.S., S.W., J.E.M., K.J.M., E.B.R., and J.E.C. participated in the interpretation of the results and critical revision of the manuscript. Y.-X.W., Y.L., and J.E.C. had full access to all the data in the study. All authors accept responsibility to submit for publication.

## Data sharing statement

Our data, including analytic code, can be available upon request with permission from our staff. Further information including the procedures for obtaining and accessing data from the Nurses’ Health Studies II is described at https://www.nurseshealthstudy.org/researchers (email: nhsaccess@channing.harvard.edu).

## Declaration of interests

E.B.R. reports financial support from USDA/United States Highbush Blueberry Council, outside the submitted work. J.E.C. reports financial support from the National Institutes of Health, US Food and Drug Administration, US Centers for Disease Control and Prevention, Harvard Health Publications, Caixa Foundation, American Society for Reproductive Medicine, Northwestern University, Pacific Coast Reproductive Society, Medical University of Vienna, outside the submitted work. All other authors declare no competing interests.

## Appendix Supplementary materials

Image, application 1
