# Supplementary material for: Associations of birth weight and later life lifestyle factors with risk of cardiovascular disease in the USA: A prospective cohort study
Source: eClinicalMedicine. 2022 Jul 18;51:101570. doi: 10.1016/j.eclinm.2022.101570 (PMC9304913; doi:10.1016/j.eclinm.2022.101570)
Supplement: Supplementary file 1 [file mmc1.pdf]

Supplemental Appendix

Associations of birth weight and later life lifestyle factors with risk of cardiovascular disease in the USA: a prospective cohort study

Yi-Xin Wang, Yanping Li, Janet W. Rich-Edwards, Andrea A. Florio, Zhilei Shan, Siwen Wang, JoAnn E. Manson, Kenneth J Mukamal, Eric B. Rimm, and Jorge E. Chavarro

**Contents**	**Pages**
Description of Cox model.	2
Figure S1. Adjusted relative risks of cardiovascular disease according to joint categories of birth weight and unhealthy lifestyle.	4
Table S1. Baseline characteristics and crude incidence of CVD of included participants versus those excluded due to missing data on birth weight.	5
Table S2. Hazard ratio (95% CI) of CHD and stroke according to birth weight among women in the Nurses' Health Study (n=52,380) and the Nurses' Health Study II (n=85,350).	6
Table S3. Multivariable-adjusted hazard ratio (95% CI) of CVD according to birth weight category among men in the Health Professionals Follow-up Study (n=20,169) and women in the Nurses' Health Study (n=52,380) and the Nurses' Health Study II (n=85,350), stratified by lifestyle factors.	8
Table S4. Attributing effects to additive interaction between birth weight and lifestyles on risks of CHD and stroke among women.	10
Table S5. Pooled adjusted attributing effects to additive interaction between birth weight and lifestyle factors on risk of CHD among women in the Nurses' Health Study (n=52,380) and the Nurses' Health Study II (n=85,350).	11
Table S6. Pooled adjusted attributing effects to additive interaction between birth weight and lifestyle factors on risk of CHD among men in the Health Professionals Follow-up Study (n=20,169).	12
Table S7. Sensitivity analysis of the association between birth weight and CVD among men in the Health Professionals Follow-up Study and women in the Nurses' Health Study and the Nurses' Health Study II, in which a missing indicator was created for participants who had missing data on lifestyle factors in multivariable Cox models.	13
Table S8. Sensitivity analysis of the association between birth weight and CVD among men in the Health Professionals Follow-up Study (n=20,169) and women in the Nurses' Health Study (n=52,380) and the Nurses' Health Study II (n=85,350), with additional adjustment for adult height.	14
Table S9. Sensitivity analysis of the association between birth weight and CVD among men in the Health Professionals Follow-up Study (n=20,169) and women in the Nurses' Health Study (n=52,380) and the Nurses' Health Study II (n=85,350), with additional adjustment for parental history of smoking status and maternal health conditions.	15
Table S10. Hazard ratio (95% CI) of CVD according to birth weight among men in the Health Professionals Follow-up Study (n=20,169) and women in the Nurses' Health Study (n=52,380) and the Nurses' Health Study II (n=85,350), with additional adjustment for socioeconomic status during infancy.	16
Table S11. Hazard ratio (95% CI) of CVD according to birth weight among men in the Health Professionals Follow-up Study (n=20,169) and women in the Nurses' Health Study (n=52,380) and the Nurses' Health Study II (n=85,350), which classified women reporting premature birth into a separate exposure category.	17
Table S12. Hazard ratio (95% CI) of CVD according to birth weight among women in the Nurses' Health Study (n=52,380) and the Nurses' Health Study II (n=85,350) by classifying the participants reporting that they were multiple births (e.g., twins and triplets) into a separate exposure category.	18
Table S13. Sensitivity analysis of attributing effects to additive interaction between birth weight and four lifestyles on the risk of CHD.	19
Table S14. Attributing effects to additive interaction between birth weight and lifestyles on risks of CHD and stroke among men in the Health Professionals Follow-up Study (n=20,169) and women in the Nurses' Health Study (n=52,380) and the Nurses' Health Study II (n=85,350).	20

Description of Cox model

The basic model for our analysis was the proportional hazards model,

$$\mathrm{I}(t, x, U, i) = I_{oi}(t) * exp(\mathbf{1} * x(t) + \mathbf{2} * U(t)),$$

where

1 is the $log_{\ e}$ of the incidence rate ratio describing the increase or decrease in the baseline incidence rate at time t due to a one-unit increase in exposure x(t) measured at time t, U(t) is a vector of other determinants of risk for the outcome at age t,

2 is the vector of $log_{\ e}$ incidence rate ratios describing the increase or decrease in the baseline incidence rate due to a one-unit increase in these other *a priori* determinants of disease risk,

t is the age at which the outcomes of interest are diagnosed, and

$I_{oi}(t)$ is the baseline incidence rate of cardiovascular disease at age t in stratum I (1,2).

Participants contributed follow-up periods from the date of returning the analysis baseline questionnaire until the date of diagnosis of cardiovascular disease, death, or end of follow-up (January 2016 in HPFS, June 2018 in NHS, and June 2017 in NHS II), whichever occurred first.

SAS PROC PHREG was used for all analyses (SAS Institute, 2004) and the Anderson-Gill data structure was used to handle time-varying covariates efficiently (3, 4). In the Anderson-Gill data structure, a new data record is created for every questionnaire cycle at which a participant was at risk, with covariates set to their values at the time the questionnaire was returned.

To control as finely as possible age, calendar time, and any possible two-way interactions between these two-time scales, we stratified the analysis jointly by age in months at the start of follow-up and calendar year of the current questionnaire cycle. The time scale for the analysis was then measured as months since the start of the current questionnaire cycle, which is equivalent to age in months because of the way we structured the data and formulate the model for analysis.

Departures from the proportional hazards assumption, i.e. effect modification by age or calendar time, were tested by likelihood ratio tests comparing models with and without the interaction terms of age or calendar time by exposure.

References:

1. Cox DR, Oakes D. Analysis of survival of survival data. London: Chapman and Hall, 1984.
2. Cox, D.R.: Regression models and life tables (with discussion). J Royal Statistical Soc: Series B, 1972; 34:187-220.
3. Therneau, TM, Extending the Cox Model, p51-84, in DY Lin and TR Fleming (editors), Proceedings of the First Seattle Symposium in Biostatistics: Survival Analysis, Springer Verlag, 1997.
4. Lin D, Fleming TR. Proceedings of the First Seattle Symposium in Biostatistics: Survival Analysis: Survival Analysis: Springer Science & Business Media, 2012.

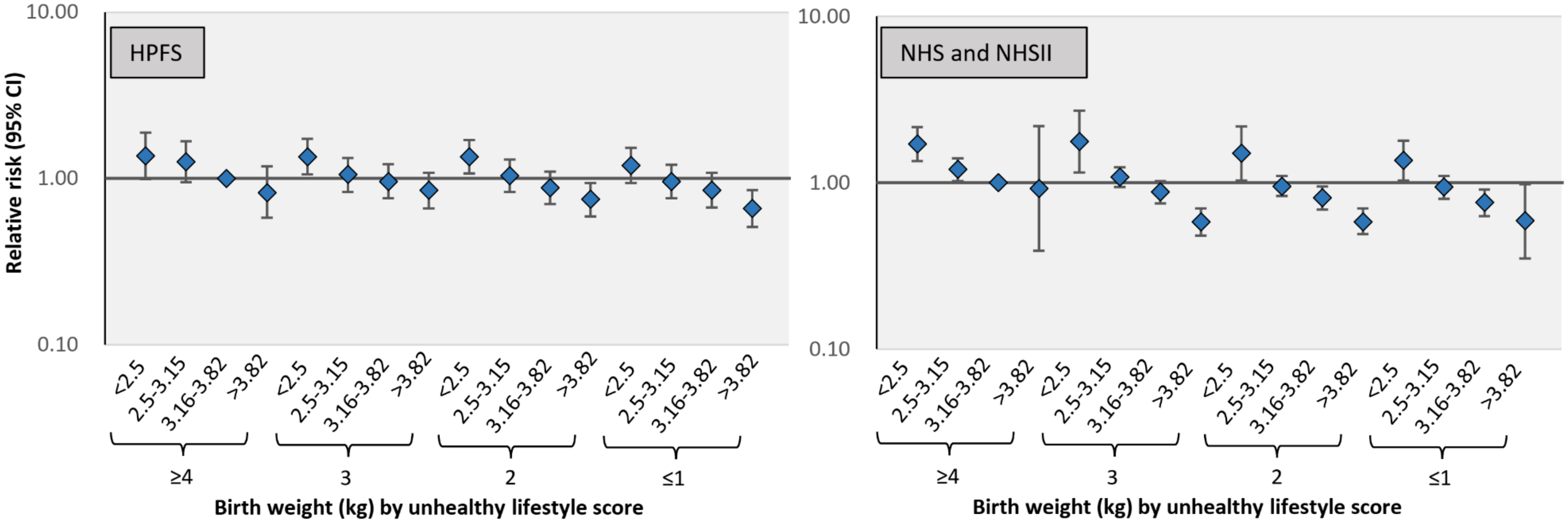

Figure S1. Adjusted relative risks of cardiovascular disease according to joint categories of birth weight and unhealthy lifestyle. Models were adjusted for age, ethnicity (white, yes/no), family history of CVD (yes/no), as well as time-varying marital status (yes/no), living status (alone or not), menopausal status [premenopausal or postmenopausal (never, past, or current menopausal hormone use), women only], smoking status (never smoker, former smoker, current smoker: 1-14, 15-24, ≥25 cigarettes/d), alcohol drinking (0, 0.1-4.9, 5.0-14.9, 15.0-19.9, 20.0-29.9, ≥30 g/d), exercise (0, 0.01-1.0, 1.0-3.5, 3.5-6.0, ≥6 h/week), DASH diet score (5 categories), and body mass index (<21, 21-24.9, 25-29.9, 30-31.9, ≥32 kg/m^2).

Table S1. Baseline characteristics and crude incidence of CVD of included participants versus those excluded due to missing data on birth weight.

Characteristics	Included participants	Excluded participants
**The Health Professionals Follow-up Study (1986)**		
Number of participants	20,169	22,876
Age (y)	51.7 (9)	54.8 (9.9)
Height (m)	1.8 (0.1)	1.8 (0.1)
BMI (kg/m^2)	25.5 (3.2)	25.4 (3.4)
Total energy intake, kcal/d	2018.8 (621.4)	1973 (618.8)
DASH diet score	24.2 (5.4)	23.8 (5.5)
Alcohol intake, g/d	11.5 (15.3)	11.2 (15.4)
Current smoking, %	8.4	9.9
Moderate to vigorous intensity exercise, h/wk	3 (4.6)	2.7 (4.1)
Crude Incidence of CVD per 100 person-years	12.01	14.03
**The Nurses' Health Study (1980)**		
Number of participants	52,380	26,151
Age (y)	45.4 (7.1)	47.5 (7.2)
Height (m)	1.6 (0.1)	1.6 (0.1)
BMI (kg/m^2)	24.3 (4.4)	24.5 (4.6)
Premenopausal, %	56.0	55.2
Total energy intake, kcal/d	1567.1 (493.9)	1563.6 (506.7)
DASH diet score	24 (4.6)	23.4 (4.7)
Alcohol intake, g/d	6.5 (10.5)	6.3 (10.7)
Current smoking, %	27.0	32.3
Moderate to vigorous intensity exercise, h/wk	4 (2.9)	3.7 (2.9)
Crude Incidence of CVD per 100 person-years	4.78	6.20
**The Nurses' Health Study II (1991)**		
Number of participants	85,350	7298
Age (y)	36 (4.7)	37.1 (4.5)
Height (m)	1.6 (0.1)	1.6 (0.1)
BMI (kg/m^2)	24.6 (5.3)	24.2 (5)
Premenopausal, %	96.4	96.4
Total energy intake, kcal/d	1792 (546.7)	1753.6 (556.8)
DASH diet score	23.6 (5)	23.2 (5)
Alcohol intake, g/d	3.1 (6.1)	3.3 (6.7)
Current smoking, %	12.2	13.3
Moderate to vigorous intensity exercise, h/wk	2.4 (3.8)	2.3 (3.8)
Crude Incidence of CVD per 100 person years	0.97	0.87

Values are means (SD) or percentages. All variables except age are age-standardized. [a]Women who reported that their menstruation had ceased as a result of surgery, radiotherapy, or chemotherapy were not categorized in the premenopausal group.

Table S2. Hazard ratio (95% CI) of CHD and stroke according to birth weight among women in the Nurses' Health Study (n=52,380) and the Nurses' Health Study II (n=85,350).

	Birthweight category (kg)					P for linear trend
	<2.5	2.5-3.15	3.16-3.82	3.83-4.5	>4.5	
**CHD**						
**The Nurses' Health Study (1980-2018)**						
Cases	708	1868	2405	600	149	-
Crude incidence, per 1000 person years	3.89	3.49	3.07	3.18	3.91	-
HR (95% CI) in age-adjusted models [a]	1.31 (1.20, 1.42)	1.15 (1.09, 1.22)	1 [Reference]	0.96 (0.88, 1.05)	1.04 (0.88, 1.22)	<0.001
HR (95% CI) in multivariable models [b]	1.29 (1.18, 1.40)	1.17 (1.10, 1.24)	1 [Reference]	0.92 (0.84, 1.01)	0.94 (0.80, 1.11)	<0.001
**The Nurses' Health Study II (1991-2017)**						
Cases	144	439	640	144	13	-
Crude incidence, per 1000 person years	0.85	0.67	0.61	0.55	0.48	-
HR (95% CI) in age-adjusted models [a]	1.31 (1.09, 1.57)	1.10 (0.97, 1.24)	1 [Reference]	0.94 (0.78, 1.12)	0.79 (0.45, 1.36)	<0.001
HR (95% CI) in multivariable models [b]	1.21 (1.01, 1.45)	1.09 (0.97, 1.23)	1 [Reference]	0.90 (0.75, 1.08)	0.71 (0.41, 1.23)	0.002
**Pooled results based on meta-analyses**						
HR (95% CI) in age-adjusted models [a]	1.31 (1.21, 1.41)	1.14 (1.08, 1.20)	1 [Reference]	0.95 (0.88, 1.03)	1.01 (0.86, 1.19)	<0.001
P for heterogeneity[c]	0.98	0.47	-	0.84	0.35	0.76
HR (95% CI) in multivariable models [b]	1.27 (1.18, 1.37)	1.15 (1.09, 1.22)	1 [Reference]	0.92 (0.85, 0.99)	0.92 (0.79, 1.08)	<0.001
P for heterogeneity[c]	0.56	0.33	-	0.85	0.34	0.77
**Stroke**						
**The Nurses' Health Study (1980-2016)**						
Cases	291	815	1142	294	70	-
Crude incidence, per 1000 person years	1.60	1.52	1.46	1.56	1.84	-
HR (95% CI) in age-adjusted models [a]	1.15 (1.01, 1.31)	1.07 (0.98, 1.17)	1 [Reference]	0.98 (0.87, 1.19)	1.00 (0.78, 1.27)	0.02
HR (95% CI) in multivariable models [b]	1.14 (1.00, 1.30)	1.07 (0.98, 1.18)	1 [Reference]	0.96 (0.85, 1.10)	0.96 (0.76, 1.23)	0.01
**The Nurses' Health Study II (1991-2017)**						
Cases	67	241	335	76	10	-
Crude incidence, per 1000 person years	0.40	0.37	0.32	0.29	0.37	-
HR (95% CI) in age-adjusted models [a]	1.18 (0.90, 1.53)	1.15 (0.97, 1.35)	1 [Reference]	0.94 (0.73, 1.20)	1.16 (0.62, 2.17)	0.07
HR (95% CI) in multivariable models [b]	1.14 (0.87, 1.48)	1.15 (0.97, 1.35)	1 [Reference]	0.91 (0.71, 1.17)	1.11 (0.59, 2.08)	0.07
**Pooled results based on meta-analyses**						
HR (95% CI) in age-adjusted models [a]	1.16 (1.03, 1.30)	1.09 (1.00, 1.17)	1 [Reference]	0.97 (0.87, 1.09)	1.02 (0.81, 1.28)	0.004
P for heterogeneity[c]	0.89	0.45	-	0.72	0.67	0.52
HR (95% CI) in multivariable models [b]	1.14 (1.02, 1.28)	1.09 (1.01, 1.18)	1 [Reference]	0.95 (0.85, 1.07)	0.98 (0.78, 1.23)	0.002
P for heterogeneity[c]	0.99	0.49	-	0.69	0.68	0.61

[a]In age-adjusted models, age in months (continuous) at the start of follow-up and calendar year of the current questionnaire cycle were included as stratified variables to control for potential confounding by age, calendar time, and any possible interactions between these two timescales. [b]Models were further adjusted for ethnicity (white, yes/no), family history of CVD (yes/no), as well as time-varying marital status (yes/no), living status (alone or not), menopausal status [premenopausal or postmenopausal (never, past, or current menopausal hormone use), women only], smoking status (never smoker, former smoker, current smoker: 1-14, 15-24, ≥25

cigarettes/d), alcohol drinking (0, 0.1-4.9, 5.0-14.9, 15.0-19.9, 20.0-29.9, ≥30 g/d), exercise (0, 0.01-1.0, 1.0-3.5, 3.5-6.0, ≥6 h/week), DASH diet score (5 categories), and body mass index (<21, 21-24.9, 25-29.9, 30-31.9, ≥32 kg/m^2). [c]Test for between-study heterogeneity.

Table S3. Multivariable-adjusted hazard ratio (95% CI) of CVD according to birth weight category among men in the Health Professionals Follow-up Study (n=20,169) and women in the Nurses' Health Study (n=52,380) and the Nurses' Health Study II (n=85,350), stratified by lifestyle factors.[a]

Lifestyle factors	Birth weight category (kg)					P for linear trend	P for interaction
	<2.5	2.5-3.15	3.16-3.82	3.83-4.5	>4.5		
**Men**							
DASH diet score							0.99
Upper two-fifths	1.17 (0.96, 1.43)	1.08 (0.97, 1.20)	1 [Reference]	0.98 (0.87, 1.10)	0.88 (0.75, 1.04)	0.005	
Bottom three-fifths	1.11 (0.96, 1.29)	1.06 (0.97, 1.15)	1 [Reference]	0.88 (0.80, 0.96)	0.93 (0.82, 1.06)	<0.001	
Physical activity at moderate intensity							0.82
≥30 minutes/day	1.06 (0.86, 1.32)	1.07 (0.96, 1.20)	1 [Reference]	0.89 (0.78, 1.01)	0.85 (0.71, 1.03)	0.004	
<30 minutes/day	1.15 (1.00, 1.33)	1.06 (0.98, 1.15)	1 [Reference]	0.91 (0.83, 1.00)	0.93 (0.83, 1.05)	<0.001	
Smoking status							0.84
Never	1.11 (0.99, 1.26)	1.06 (0.99, 1.13)	1 [Reference]	0.90 (0.84, 0.98)	0.90 (0.81, 1.00)	<0.001	
Former/current smokers	1.25 (0.78, 1.99)	1.13 (0.87, 1.48)	1 [Reference]	0.88 (0.65, 1.19)	1.20 (0.82, 1.75)	0.38	
BMI							0.009
<25 kg/m^2	1.21 (1.00, 1.45)	1.13 (1.02, 1.26)	1 [Reference]	0.90 (0.79, 1.02)	0.75 (0.62, 0.91)	<0.001	
≥25 kg/m^2	1.08 (0.92, 1.26)	1.02 (0.94, 1.11)	1 [Reference]	0.91 (0.84, 1.00)	1.00 (0.88, 1.12)	0.07	
Moderate alcohol consumption							0.89
Yes	1.21 (1.00, 1.47)	1.11 (1.00, 1.23)	1 [Reference]	0.93 (0.83, 1.05)	0.96 (0.81, 1.12)	0.002	
No	1.10 (0.95, 1.28)	1.04 (0.95, 1.13)	1 [Reference]	0.89 (0.81, 0.98)	0.87 (0.77, 0.99)	<0.001	
**Women**							
DASH diet score							0.86
Upper two-fifths	1.26 (1.13, 1.40)	1.06 (0.91, 1.22)	1 [Reference]	0.88 (0.77, 1.01)	0.95 (0.77, 1.18)	<0.001	
Bottom three-fifths	1.22 (1.13, 1.32)	1.16 (1.10, 1.23)	1 [Reference]	0.96 (0.89, 1.05)	0.91 (0.77, 1.08)	<0.001	
Physical activity at moderate intensity							0.47
≥30 minutes/day	1.37 (1.15, 1.64)	1.20 (1.06, 1.35)	1 [Reference]	0.98 (0.75, 1.28)	1.09 (0.77, 1.55)	<0.001	
<30 minutes/day	1.20 (1.09, 1.33)	1.13 (1.08, 1.18)	1 [Reference]	0.93 (0.85, 1.01)	0.90 (0.78, 1.04)	<0.001	
Smoking status							0.58
Never	1.23 (1.15, 1.32)	1.13 (1.06, 1.19)	1 [Reference]	0.95 (0.88, 1.02)	0.92 (0.80, 1.06)	<0.001	
Former/current smokers	1.14 (0.84, 1.54)	1.15 (0.99, 1.33)	1 [Reference]	0.86 (0.73, 1.01)	1.03 (0.75, 1.41)	<0.001	
BMI							0.87
<25 kg/m^2	1.24 (1.12, 1.37)	1.09 (1.02, 1.17)	1 [Reference]	0.89 (0.80, 0.99)	0.99 (0.80, 1.24)	<0.001	
≥25 kg/m^2	1.23 (1.14, 1.34)	1.15 (1.09, 1.22)	1 [Reference]	0.97 (0.90, 1.06)	0.92 (0.78, 1.08)	<0.001	
Moderate alcohol consumption							0.23
Yes	1.25 (1.06, 1.48)	0.99 (0.83, 1.18)	1 [Reference]	1.03 (0.75, 1.41)	1.09 (0.79, 1.51)	0.13	
No	1.26 (1.18, 1.35)	1.17 (1.11, 1.23)	1 [Reference]	0.93 (0.85, 1.02)	0.92 (0.80, 1.06)	<0.001	
**Pooled results based on meta-analyses**							
DASH diet score							0.88
Upper two-fifths	1.24 (1.13, 1.36)	1.08 (1.01, 1.16)	1 [Reference]	0.92 (0.84, 1.01)	0.91 (0.80, 1.03)	<0.001	

Bottom three-fifths	1.19 (1.11, 1.28)	1.13 (1.06, 1.21)	1 [Reference]	0.93 (0.87, 0.99)	0.92 (0.83, 1.02)	<0.001	
Physical activity at moderate intensity							0.51
≥30 minutes/day	1.27 (1.03, 1.55)	1.13 (1.04, 1.23)	1 [Reference]	0.92 (0.81, 1.04)	0.90 (0.76, 1.06)	<0.001	
<30 minutes/day	1.20 (1.13, 1.28)	1.11 (1.06, 1.16)	1 [Reference]	0.93 (0.88, 0.98)	0.92 (0.84, 1.01)	<0.001	
Smoking status							0.56
Never	1.20 (1.13, 1.28)	1.10 (1.03, 1.16)	1 [Reference]	0.93 (0.88, 0.98)	0.91 (0.83, 0.99)	<0.001	
Former/current smokers	1.19 (0.99, 1.43)	1.13 (1.03, 1.25)	1 [Reference]	0.86 (0.75, 1.00)	1.09 (0.86, 1.39)	<0.001	
BMI							0.38
<25 kg/m^2	1.23 (1.12, 1.34)	1.10 (1.04, 1.17)	1 [Reference]	0.89 (0.82, 0.97)	0.87 (0.69, 1.09)	<0.001	
≥25 kg/m^2	1.18 (1.08, 1.30)	1.11 (1.02, 1.20)	1 [Reference]	0.95 (0.89, 1.01)	0.97 (0.88, 1.07)	<0.001	
Moderate alcohol consumption							0.29
Yes	1.24 (1.09, 1.40)	1.05 (0.95, 1.17)	1 [Reference]	0.96 (0.84, 1.10)	0.98 (0.85, 1.14)	<0.001	
No	1.21 (1.10, 1.33)	1.12 (1.04, 1.21)	1 [Reference]	0.92 (0.86, 0.98)	0.89 (0.81, 0.98)	<0.001	

[a]Cox proportional hazards models were adjusted for age, ethnicity (white, yes/no), family history of CVD (yes/no), as well as time-varying marital status (yes/no), living status (alone or not), menopausal status [premenopausal or postmenopausal (never, past, or current menopausal hormone use), women only]. [b]Unhealthy lifestyles include currently smoking, exercising <30 min/d at moderate intensity, DASH diet score in the bottom three fifths, body mass index ≥25, and not moderate alcohol consumption (moderate: 5-15 g alcohol/d in women).

Table S4. Attributing effects to additive interaction between birth weight and lifestyles on risks of CHD and stroke among women.[a]

	The Nurses' Health Study	The Nurses' Health Study II	Pooled results	P for heterogeneity[c]
**CHD**				
Main effects				
Lower birth weight (per kg)	1.13 (1.02, 1.26)	1.12 (0.88, 1.43)	1.13 (1.02, 1.25)	0.94
Time-varing unhealthy lifestyles score (1-4)[b]	1.33 (1.23, 1.44)	1.52 (1.29, 1.80)	1.39 (1.23, 1.58)	0.15
Joint effect	1.52 (1.38, 1.65)	1.73 (1.42, 2.03)	1.58 (1.41, 1.78)	0.21
Measures of interaction				
Relative excess risk due to interaction	0.06 (0.03, 0.08)	0.08 (0.03, 0.14)	0.06 (0.04, 0.08)	0.43
P for additive interaction	<0.001	0.004	<0.001	0.41
P for multiplicative interaction	0.68	0.82	0.64	0.97
Attributable proportion, %				
Lower birth weight	25.3% (11.6, 39.1%)	16.5% (-9.4, 42.5%)	23.0% (11.0, 36.0%)	0.56
Unhealthy lifestyles[b]	63.5% (57.0, 70.0%)	72.1% (61.2, 82.9%)	67.0% (58.0, 75.0%)	0.18
Additive interaction	11.2% (3.4, 19.0%)	11.4% (1.5, 21.4%)	11.0% (5.0, 17.0%)	0.97
**Stroke**				
Main effects				
Lower birth weight (per kg)	1.07 (0.92, 1.25)	1.19 (0.88, 1.59)	1.10 (0.96, 1.25)	0.56
Time-varing unhealthy lifestyles score (1-4)[b]	1.23 (1.10, 1.38)	1.37 (1.10, 1.70)	1.26 (1.14, 1.39)	0.38
Joint effect	1.32 (1.13, 1.51)	1.58 (1.21, 1.95)	1.41 (1.19, 1.66)	0.21
Measures of interaction				
Relative excess risk due to interaction	0.02 (-0.03, 0.07)	0.02 (-0.06, 0.10)	0.02 (-0.02, 0.06)	0.96
P for additive interaction	0.43	0.59	0.67	0.96
P for multiplicative interaction	0.96	0.66	0.88	0.68
Attributable proportion, %				
Lower birth weight	22.9% (-10.4, 56.2%)	31.9% (2.1, 61.8%)	28.0% (6.0, 50.0%)	0.69
Unhealthy lifestyles[b]	71.1% (57.4, 84.8%)	64.3% (48.4, 80.2%)	67.0% (58.0, 79.0%)	0.53
Additive interaction	6.0 % (-12.7, 24.7%)	3.8% (-11.9, 19.4%)	5.0% (-7.0, 17.0%)	0.86

[a]Cox proportional hazards models were adjusted for age, ethnicity (white, yes/no), family history of CVD (yes/no), as well as time-varying marital status (yes/no), living status (alone or not), menopausal status [premenopausal or postmenopausal (never, past, or current menopausal hormone use), women only]. [b]Unhealthy lifestyles include currently smoking, exercising <30 min/d at moderate intensity, DASH diet score in the bottom three fifths, body mass index ≥25, and not moderate alcohol consumption (moderate: 5-15 g alcohol/d in women). [c]Test for between-study heterogeneity.

Table S5. Pooled adjusted attributing effects to additive interaction between birth weight and lifestyle factors on risk of CHD among women in the Nurses' Health Study (n=52,380) and the Nurses' Health Study II (n=85,350).[a]

Attributing effects	Estimations (95% CI)				
	**BMI[b]**	**Smoking status[c]**	**DASH diet score[d]**	**Physical activity[e]**	**Alcohol intake[f]**
Main effects					
Lower birth weight (per kg)	1.14 (1.08, 1.22)	1.15 (1.10, 1.19)	1.15 (1.08, 1.22)	1.19 (1.08, 1.32)	1.08 (0.98, 1.19)
Time-varing unhealthy lifestyles	1.48 (1.29, 1.69)	2.49 (1.59, 3.88)	1.36 (1.01, 1.81)	1.56 (1.29, 1.88)	1.13 (0.94, 1.34)
Joint effect	1.79 (1.55, 2.06)	2.91 (1.93, 4.39)	1.58 (1.20, 2.08)	1.78 (1.58, 2.00)	1.30 (1.15, 1.48)
Measures of interaction					
9 Relative excess risk due to interaction	0.15 (-0.02, 0.33)	0.19 (0.02, 0.36)	0.04 (-0.04, 0.11)	0.03 (-0.08, 0.13)	0.10 (0.01, 0.19)
P for additive interaction	0.09	0.03	0.34	0.62	0.03
P for multiplicative interaction	0.55	0.89	0.95	0.42	0.19
Attributable proportion, %					
Lower birth weight	16.0% (0, 32.0%)	8.0% (4.0, 13.0%)	30.0% (18.0, 42.0%)	25.0% (17.0, 32.0%)	29.0% (11.0, 48.0%)
Unhealthy lifestyles[b]	66.0% (56.0, 75.0%)	78.0% (68.0, 89.0%)	62.0% (40.0, 84.0%)	71.0% (58.0, 84.0%)	41.0% (26.0, 57.0%)
Additive interaction	15.0% (1.0, 28.0%)	12.0% (0, 25.0%)	6.0 % (-12.0, 24.0%)	3.0% (-11.0, 17.0%)	30.0% (-19.0, 79.0%)

[a]Cox proportional hazards models were adjusted for age, ethnicity (white, yes/no), family history of CVD (yes/no), as well as time-varying marital status (yes/no), living status (alone or not), menopausal status [premenopausal or postmenopausal (never, past, or current menopausal hormone use), women only]. [b]BMI was tested using categorical variables (<25 vs. ≥25 kg/m^2). [c]Smoking status was tested using dichotomous variables (current vs. non-current smokers). [d]Dietary quality was tested using dichotomous variables (the bottom three-fifths vs. the upper two-fifths of the DASH diet score). [e]Physical activity was tested using dichotomous variables (<30 vs. ≥30 min/day at moderate intensity). [f]Alcohol consumption was tested using dichotomous variables (moderate: 5-15 g alcohol/d vs. non-moderate).

Table S6. Adjusted attributing effects to additive interaction between birth weight and lifestyle factors on risk of CHD among men in the Health Professionals Follow-up Study (n=20,169).[a]

**Attributing effects**	**Estimations (95% CI)**				
	**BMI[b]**	**Smoking status[c]**	**DASH diet score[d]**	**Physical activity[e]**	**Alcohol intake[f]**
Main effects					
Lower birth weight (per kg)	1.19 (1.10, 1.28)	1.10 (1.05, 1.15)	1.08 (1.01, 1.16)	1.12 (1.04, 1.21)	1.01 (0.80, 1.29)
Time-varing unhealthy lifestyles	1.56 (1.36, 1.78)	1.61 (1.25, 2.07)	1.13 (0.99, 1.29)	1.31 (1.14, 1.50)	1.11 (0.73, 1.69)
Joint effect	1.66 (1.54, 1.77)	2.91 (1.93, 4.39)	1.24 (1.13, 1.35)	1.42 (1.30, 1.54)	1.30 (0.90, 1.70)
Measures of interaction					
Relative excess risk due to interaction	-0.09 (-0.20, 0.02)	0.02 (-0.25, 0.29)	0.03 (-0.05, 0.12)	-0.01 (-0.11, 0.09)	0.18 (-0.02, 0.38)
P for additive interaction	0.13	0.89	0.44	0.84	0.08
P for multiplicative interaction	0.02	0.80	0.66	0.49	0.27
Attributable proportion, %					
Lower birth weight	28.0% (21.0, 35.7%)	13.0% (7.0, 20.0%)	32.0% (14.0, 51.0%)	28.0% (16.0, 41.0%)	5.0% (-69.0, 78.0%)
Unhealthy lifestyles[b]	85.0% (71.0, 98.0%)	84.0% (59.0, 109.0%)	53.0% (35.0, 71.0%)	74.0% (58.0, 90.0%)	36.0% (-4.0, 76.0%)
Additive interaction	-13.0% (-28.0, 2.0%)	3.0% (-35.0, 40.0%)	14.0 % (-28.0, 56.0%)	-2.0% (-26.0, 21.0%)	60.0% (-101.0, 220.0%)

[a]Cox proportional hazards models were adjusted for age, ethnicity (white, yes/no), family history of CVD (yes/no), as well as time-varying marital status (yes/no), living status (alone or not), menopausal status [premenopausal or postmenopausal (never, past, or current menopausal hormone use), women only]. [b]BMI was tested using categorical variables (<25 vs. ≥25 kg/m^2). [c]Smoking status was tested using dichotomous variables (current vs. non-current smokers). [d]Dietary quality was tested using dichotomous variables (the bottom three-fifths vs. the upper two-fifths of the DASH diet score). [e]Physical activity was tested using dichotomous variables (<30 vs. ≥30 min/day at moderate intensity). [f]Alcohol consumption was tested using dichotomous variables (moderate: 5-15 g alcohol/d vs. non-moderate).

Table S7. Sensitivity analysis of the association between birth weight and CVD among men in the Health Professionals Follow-up Study and women in the Nurses' Health Study and the Nurses' Health Study II, in which a missing indicator was created for participants who had missing data on lifestyle factors in multivariable Cox models.

CVD	Birthweight category (kg)					P for non-linear trend	P for linear trend
	<2.5	2.5-3.15	3.16-3.82	3.83-4.5	>4.5		
**The Health Professionals Follow-up Study (1986-2016)**							
CVD cases	348	1508	2950	1102	515		
Crude incidence, per 1000 person years	14.25	12.76	11.97	11.91	14.05		
HR (95% CI) in age-adjusted models [a]	1.16 (1.04, 1.30)	1.07 (1.00, 1.14)	1 [Reference]	0.95 (0.88, 1.01)	0.93 (0.84, 1.02)	0.47	<0.001
HR (95% CI) in multivariable models [b]	1.13 (1.01, 1.27)	1.08 (1.01, 1.14)	1 [Reference]	0.92 (0.86, 0.99)	0.89 (0.81, 0.98)	0.65	<0.001
**The Nurses' Health Study (1980-2018)**							
CVD cases	1419	3645	4809	1241	299		
Crude incidence, per 1000 person years	5.66	5.07	4.62	4.90	5.68		
HR (95% CI) in age-adjusted models [a]	1.25 (1.18, 1.33)	1.12 (1.07, 1.17)	1 [Reference]	0.98 (0.92, 1.05)	0.99 (0.88, 1.12)	0.09	<0.001
HR (95% CI) in multivariable models [b]	1.24 (1.17, 1.32)	1.13 (1.09, 1.18)	1 [Reference]	0.96 (0.90, 1.02)	0.93 (0.83, 1.05)	0.67	<0.001
**The Nurses' Health Study II (1991-2017)**							
CVD cases	224	728	1028	238	26		
Crude incidence, per 1000 person years	1.24	1.04	0.93	0.85	0.89		
HR (95% CI) in age-adjusted models [a]	1.25 (1.09, 1.45)	1.12 (1.02, 1.23)	1 [Reference]	0.96 (0.83, 1.11)	0.96 (0.65, 1.41)	0.63	0.001
HR (95% CI) in multivariable models [b]	1.18 (1.02, 1.36)	1.12 (1.02, 1.23)	1 [Reference]	0.93 (0.80, 1.07)	0.88 (0.59, 1.29)	0.69	<0.001

[a]In age-adjusted models, age in months (continuous) at the start of follow-up and calendar year of the current questionnaire cycle were included as stratified variables to control for potential confounding by age, calendar time, and any possible interactions between these two timescales. [b]Models were further adjusted for ethnicity (white, yes/no), family history of CVD (yes/no), as well as time-varying marital status (yes/no), menopausal status [premenopausal or postmenopausal (never, past, or current menopausal hormone use), women only], smoking status (never smoker, former smoker, current smoker: 1-14, 15-24, ≥25 cigarettes/d), alcohol drinking (0, 0.1-4.9, 5.0-14.9, 15.0-19.9, 20.0-29.9, ≥30 g/d), exercise (0, 0.01-1.0, 1.0-3.5, 3.5-6.0, ≥6 h/week), DASH diet score (5 categories), and body mass index (<21, 21-24.9, 25-29.9, 30-31.9, ≥32 kg/m^2).

Table S8. Sensitivity analysis of the association between birth weight and CVD among men in the Health Professionals Follow-up Study (n=20,169) and women in the Nurses' Health Study (n=52,380) and the Nurses' Health Study II (n=85,350), with additional adjustment for adult height.

CVD	Birthweight category (kg)					P for non-linear trend	P for linear trend
	<2.5	2.5-3.15	3.16-3.82	3.83-4.5	>4.5		
**The Health Professionals Follow-up Study (1986-2016)**							
CVD cases	313	1349	2659	969	460		
Crude incidence, per 1000 person years	13.73	12.30	11.65	11.37	13.89		
HR (95% CI) in age-adjusted models [a]	1.16 (1.03, 1.30)	1.06 (0.99, 1.13)	1 [Reference]	0.93 (0.87, 1.00)	0.94 (0.85, 1.04)	0.50	<0.001
HR (95% CI) in multivariable models [b]	1.11 (0.99, 1.25)	1.05 (0.99, 1.13)	1 [Reference]	0.91 (0.85, 0.98)	0.92 (0.83, 1.02)	0.67	<0.001
**The Nurses' Health Study (1980-2018)**							
CVD cases	989	2652	3502	888	213		
Crude incidence, per 1000 person years	5.45	4.96	4.47	4.71	5.60		
HR (95% CI) in age-adjusted models [a]	1.26 (1.17, 1.35)	1.13 (1.07, 1.19)	1 [Reference]	0.97 (0.90, 1.05)	1.01 (0.88, 1.16)	0.27	<0.001
HR (95% CI) in multivariable models [b]	1.24 (1.16, 1.34)	1.14 (1.09, 1.20)	1 [Reference]	0.94 (0.88, 1.02)	0.93 (0.81, 1.07)	0.99	<0.001
**The Nurses' Health Study II (1991-2017)**							
CVD cases	209	676	968	220	23		
Crude incidence, per 1000 person years	1.24	1.03	0.92	0.83	0.85		
HR (95% CI) in age-adjusted models [a]	1.26 (1.09, 1.46)	1.12 (1.01, 1.23)	1 [Reference]	0.94 (0.82, 1.09)	0.92 (0.61, 1.39)	0.72	<0.001
HR (95% CI) in multivariable models [b]	1.19 (1.02, 1.38)	1.11 (1.01, 1.23)	1 [Reference]	0.91 (0.79, 1.06)	0.85 (0.56, 1.29)	0.64	<0.001

[a]In age-adjusted models, age in months (continuous) at the start of follow-up and calendar year of the current questionnaire cycle were included as stratified variables to control for potential confounding by age, calendar time, and any possible interactions between these two timescales. [b]Models were further adjusted for ethnicity (white, yes/no), adult height (continuous), family history of CVD (yes/no), as well as time-varying marital status (yes/no), menopausal status [premenopausal or postmenopausal (never, past, or current menopausal hormone use), women only], smoking status (never smoker, former smoker, current smoker: 1-14, 15-24, ≥25 cigarettes/d), alcohol drinking (0, 0.1-4.9, 5.0-14.9, 15.0-19.9, 20.0-29.9, ≥30 g/d), exercise (0, 0.01-1.0, 1.0-3.5, 3.5-6.0, ≥6 h/week), DASH diet score (5 categories), and body mass index (<21, 21-24.9, 25-29.9, 30-31.9, ≥32 kg/m^2).

Table S9. Sensitivity analysis of the association between birth weight and CVD among men in the Health Professionals Follow-up Study (n=20,169) and women in the Nurses' Health Study (n=52,380) and the Nurses' Health Study II (n=85,350), with additional adjustment for parental history of smoking status and maternal health conditions.

CVD	Birthweight category (kg)					P for non-linear trend	P for linear trend[b]
	**<2.5**	**2.5-3.15**	**3.16-3.82**	**3.83-4.5**	**>4.5**		
**The Health Professionals Follow-up Study (1986-2016)**							
CVD cases	313	1349	2659	969	460		
Crude incidence, per 1000 person years	13.73	12.30	11.65	11.37	13.89		
HR (95% CI) in multivariable models [a]	1.12 (0.99, 1.26)	1.06 (1.00, 1.14)	1 [Reference]	0.91 (0.84, 0.97)	0.91 (0.82, 1.00)	0.64	<0.001
**The Nurses' Health Study (1980-2018)**							
CVD cases	989	2652	3502	888	213		
Crude incidence, per 1000 person years	5.45	4.96	4.47	4.71	5.60		
HR (95% CI) in multivariable models [a]	1.24 (1.16, 1.33)	1.14 (1.08, 1.20)	1 [Reference]	0.94 (0.87, 1.01)	0.93 (0.81, 1.07)	0.91	<0.001
**The Nurses' Health Study II (1991-2017)**							
CVD cases	209	676	968	220	23		
Crude incidence, per 1000 person years	1.24	1.03	0.92	0.83	0.85		
HR (95% CI) in multivariable models [a]	1.18 (1.01, 1.37)	1.11 (1.01, 1.22)	1 [Reference]	0.91 (0.79, 1.05)	0.84 (0.56, 1.28)	0.61	<0.001

[a]Models were adjusted for age, ethnicity (White, yes/no), currently married (yes/no), family history of CVD (yes/no), maternal history of diabetes (yes/no), maternal history of hypertension (yes/no), parental history of smoking during childhood (yes/no), as well as time-varying marital status (yes/no), menopausal status [premenopausal or postmenopausal (never, past, or current menopausal hormone use), women only], smoking status (never smoker, former smoker, current smoker: 1-14, 15-24, ≥25 cigarettes/d), alcohol drinking (0, 0.1-4.9, 5.0-14.9, 15.0-19.9, 20.0-29.9, ≥30 g/d), exercise (0, 0.01-1.0, 1.0-3.5, 3.5-6.0, ≥6 h/week), DASH diet score (5 categories), and body mass index (<21, 21-24.9, 25-29.9, 30-31.9, ≥32 kg/m^2). [b]Tests for linear trends were conducted by modeling birthweight categories as an ordinal level variable by assigning the median value to each category, excluding women reporting premature birth.

Table S10. Hazard ratio (95% CI) of CVD according to birth weight among men in the Health Professionals Follow-up Study (n=20,169) and women in the Nurses' Health Study (n=52,380) and the Nurses' Health Study II (n=85,350), with additional adjustment for socioeconomic status during infancy.

CVD	Birthweight category (kg)					P for non-linear trend	P for linear trend[b]
	**<2.5**	**2.5-3.15**	**3.16-3.82**	**3.83-4.5**	**>4.5**		
**The Nurses' Health Study (1980-2018)**							
CVD cases	989	2652	3502	888	213		
Crude incidence, per 1000 person years	5.45	4.96	4.47	4.71	5.60		
HR (95% CI) in multivariable models [a]	1.24 (1.15, 1.33)	1.14 (1.08, 1.20)	1 [Reference]	0.94 (0.87, 1.01)	0.94 (0.81, 1.07)	0.95	<0.001
**The Nurses' Health Study II (1991-2017)**							
CVD cases	209	676	968	220	23		
Crude incidence, per 1000 person years	1.24	1.03	0.92	0.83	0.85		
HR (95% CI) in multivariable models [a]	1.19 (1.02, 1.38)	1.12 (1.01, 1.23)	1 [Reference]	0.91 (0.79, 1.05)	0.85 (0.56, 1.28)	0.63	<0.001

[a]Models were adjusted for age, ethnicity (White, yes/no), parents owned home at the time of birth or during infancy (yes/no), parents worked as a professional, manager, or executive during infancy (yes/no), family history of CVD (yes/no), as well as time-varying marital status (yes/no), living status (alone or not), menopausal status [premenopausal or postmenopausal (never, past or current menopausal hormone use), women only], smoking status (never smoker, former smoker, current smoker: 1-14, 15-24, ≥25 cigarettes/d), alcohol drinking (0, 0.1-4.9, 5.0-14.9, 15.0-19.9, 20.0-29.9, ≥30 g/d), exercise (0, 0.01-1.0, 1.0-3.5, 3.5-6.0, ≥6 h/week), DASH diet score (5 categories), and body mass index (<21, 21-24.9, 25-29.9, 30-31.9, ≥32 kg/m^2). [b]Tests for linear trends were conducted by modeling birthweight categories as an ordinal level variable by assigning the median value to each category, excluding women reporting premature birth.

Table S11. Hazard ratio (95% CI) of CVD according to birth weight among men in the Health Professionals Follow-up Study (n=20,169) and women in the Nurses' Health Study (n=52,380) and the Nurses' Health Study II (n=85,350), which classified women reporting premature birth into a separate exposure category.

CVD	Preterm birth	Birthweight category (kg)					P for linear trend[c]
		<2.5	2.5-3.15	3.16-3.82	3.83-4.5	>4.5	
**The Nurses' Health Study (1980-2018)**							
CVD cases	441	636	2593	3478	886	210	
Crude incidence, per 1000 person years	5.14	5.43	4.99	4.47	4.72	5.55	
HR (95% CI) in age-adjusted models [a]	1.22 (1.11, 1.35)	1.25 (1.15, 1.36)	1.13 (1.08, 1.19)	1 [Reference]	0.98 (0.91, 1.05)	1.00 (0.87, 1.15)	<0.001
HR (95% CI) in multivariable models [b]	1.19 (1.08, 1.32)	1.24 (1.14, 1.35)	1.15 (1.09, 1.21)	1 [Reference]	0.94 (0.88, 1.02)	0.93 (0.81, 1.07)	<0.001
**The Nurses' Health Study II (1991-2017)**							
CVD cases	195	106	601	953	218	23	
Crude incidence, per 1000 person years	1.14	1.30	1.01	0.93	0.83	0.86	
HR (95% CI) in age-adjusted models [a]	1.26 (1.08, 1.47)	1.30 (1.06, 1.58)	1.09 (0.98, 1.20)	1 [Reference]	0.94 (0.81, 1.09)	0.93 (0.61, 1.40)	0.003
HR (95% CI) in multivariable models [b]	1.21 (1.04, 1.41)	1.20 (0.98, 1.47)	1.09 (0.98, 1.20)	1 [Reference]	0.91 (0.79, 1.06)	0.86 (0.57, 1.30)	0.003

[a]In age-adjusted models, age in months (continuous) at the start of follow-up and calendar year of the current questionnaire cycle were included as stratified variables. [b]Models were further adjusted for ethnicity (white, yes/no), currently married (yes/no), living status (alone or not), family history of CVD (yes/no), menopausal status [premenopausal or postmenopausal (never, past, or current menopausal hormone use), women only], smoking status (never smoker, former smoker, current smoker: 1-14, 15-24, ≥25 cigarettes/d), alcohol drinking (0, 0.1-4.9, 5.0-14.9, 15.0-19.9, 20.0-29.9, ≥30 g/d), exercise (0, 0.01-1.0, 1.0-3.5, 3.5-6.0, ≥6 h/week), DASH diet score (5 categories), and body mass index (<21, 21-24.9, 25-29.9, 30-31.9, ≥32). [c]Tests for linear trends were conducted by modeling birthweight categories as an ordinal level variable by assigning the median value to each category, excluding women reporting premature birth.

Table S12. Hazard ratio (95% CI) of CVD according to birth weight among women in the Nurses' Health Study (n=52,380) and the Nurses' Health Study II (n=85,350) by classifying the participants reporting that they were multiple births (e.g., twins and triplets) into a separate exposure category.

CVD	Multiple births	Birthweight category (kg)					P for linear trend[c]
		<2.5	2.5-3.15	3.16-3.82	3.83-4.5	>4.5	
**The Nurses' Health Study (1980-2018)**							
CVD cases	153	898	2614	3480	886	213	
Crude incidence, per 1000 person years	5.04	5.48	4.96	4.46	4.71	5.60	
HR (95% CI) in age-adjusted models [a]	1.15 (0.98, 1.35)	1.27 (1.18, 1.37)	1.13 (1.07, 1.19)	1 [Reference]	0.97 (0.90, 1.05)	1.01 (0.88, 1.16)	<0.001
HR (95% CI) in multivariable models [b]	1.18 (1.00, 1.39)	1.25 (1.16, 1.35)	1.14 (1.09, 1.20)	1 [Reference]	0.94 (0.88, 1.01)	0.94 (0.82, 1.08)	<0.001
**The Nurses' Health Study II (1991-2017)**							
CVD cases	33	191	661	968	220	23	
Crude incidence, per 1000 person years	0.93	1.27	1.03	0.93	0.84	0.85	
HR (95% CI) in age-adjusted models [a]	0.99 (0.70, 1.41)	1.29 (1.10, 1.51)	1.11 (1.00, 1.22)	1 [Reference]	0.94 (0.81, 1.09)	0.92 (0.61, 1.39)	<0.001
HR (95% CI) in multivariable models [b]	1.00 (0.71, 1.42)	1.21 (1.03, 1.41)	1.11 (1.00, 1.22)	1 [Reference]	0.91 (0.78, 1.05)	0.85 (0.56, 1.28)	<0.001

[a]In age-adjusted models, age in months (continuous) at the start of follow-up and calendar year of the current questionnaire cycle were included as stratified variables to control for potential confounding by age, calendar time, and any possible interactions between these two timescales. [b]Models were further adjusted for ethnicity (white, yes/no), family history of CVD (yes/no), as well as time-varying marital status (yes/no), menopausal status [premenopausal or postmenopausal (never, past, or current menopausal hormone use), women only], smoking status (never smoker, former smoker, current smoker: 1-14, 15-24, ≥25 cigarettes/d), alcohol drinking (0, 0.1-4.9, 5.0-14.9, 15.0-19.9, 20.0-29.9, ≥30 g/d), exercise (0, 0.01-1.0, 1.0-3.5, 3.5-6.0, ≥6 h/week), DASH diet score (5 categories), and body mass index (<21, 21-24.9, 25-29.9, 30-31.9, ≥32 kg/m^2). [c]Tests for linear trends were conducted by modeling birthweight categories as an ordinal level variable by assigning the median value to each category, excluding women reporting premature birth.

Table S13. Sensitivity analysis of attributing effects to additive interaction between birth weight and four lifestyles on the risk of CHD.[a]

	The Nurses' Health Study	The Nurses' Health Study II	Pooled results	P for heterogeneity[c]
Main effects				
Lower birth weight (per kg)	1.14 (1.03, 1.26)	1.16 (0.94, 1.42)	1.14 (1.05, 1.25)	0.92
Time-varing unhealthy lifestyles score[b]	1.33 (1.20, 1.47)	1.53 (1.23, 1.89)	1.38 (1.22, 1.55)	0.25
Joint effect	1.52 (1.39, 1.66)	1.75 (1.47, 2.04)	1.61 (1.40, 1.84)	0.13
Measures of interaction				
Relative excess risk due to interaction	0.05 (0.01, 0.09)	0.07 (-0.02, 0.16)	0.05 (0.02, 0.09)	0.68
P for additive interaction	0.02	0.12	0.007	0.68
P for multiplicative interaction	0.93	0.93	0.97	0.90
Attributable proportion, %				
Lower birth weight	27.1% (15.4, 38.8%)	20.6% (1.5, 39.6%)	25.0% (15.0, 35.0%)	0.59
Unhealthy lifestyles[b]	63.2% (55.5, 70.9%)	70.0% (56.4, 83.6%)	65.0% (58.0, 72.0%)	0.42
Additive interaction	9.7% (-1.7, 21.1%)	9.5% (-6.3, 25.2%)	10.0% (0.1, 19.0%)	0.98

[a]Cox proportional hazards models were adjusted for age, ethnicity (white, yes/no), family history of CVD (yes/no), as well as time-varying marital status (yes/no), living status (alone or not), menopausal status [premenopausal or postmenopausal (never, past, or current menopausal hormone use), women only]. [b]Unhealthy lifestyles include currently smoking, exercising <30 min/d at moderate intensity, DASH diet score in the bottom three fifths, and not moderate alcohol consumption (moderate: 5-15 g alcohol/d in women). [c]Test for between-study heterogeneity.

Table S14. Attributing effects to additive interaction between birth weight and lifestyles on risks of CHD and stroke among men in the Health Professionals Follow-up Study (n=20,169) and women in the Nurses' Health Study (n=52,380) and the Nurses' Health Study II (n=85,350). *

	Men	Women	Pooled results	P for heterogeneity‡
**CHD**				
Main effects				
Lower birth weight (per kg)	1.13 (1.05 to 1.21)	1.13 (1.05 to 1.22)	1.13 (1.07 to 1.19)	0.99
Time-varing unhealthy lifestyles score (1-4) †	1.29 (1.19 to 1.39)	1.42 (1.13 to 1.77)	1.34 (1.21 to 1.49)	0.04
Joint effect	1.41 (1.33 to 1.50)	1.64 (1.31 to 2.05)	1.54 (1.37 to 1.75)	0.002
Measures of interaction				
Relative excess risk due to interaction	0.004 (-0.05 to 0.06)	0.07 (0.03 to 0.10)	0.05 (0.01 to 0.09)	0.10
P for additive interaction	0.88	<0.001	0.04	0.07
P for multiplicative interaction	0.41	0.52	0.99	0.56
Attributable proportion, %				
Lower birth weight	30.4% (19.7 to 41.2%)	24.0% (15.0 to 41.0%)	27.0% (20.0 to 34.0%)	0.38
Unhealthy lifestyles	68.6% (60.0 to 77.2%)	65.0% (53.0 to 77.0%)	66.0% (58.0 to 74.0%)	0.08
Additive interaction	0.9% (-11.8 to 13.7%)	12.0% (4.0 to 20.0%)	9.0% (2.0 to 16.0%)	0.34
**Stroke**				
Main effects				
Lower birth weight (per kg)	1.09 (0.92 to 1.29)	1.09 (0.98 to 1.20)	1.09 (1.00 to 1.19)	0.92
Time-varing unhealthy lifestyles score (1-4) †	1.35 (1.12 to 1.63)	1.25 (1.13 to 1.39)	1.28 (1.17 to 1.40)	0.64
Joint effect	1.38 (1.16 to 1.59)	1.38 (1.23 to 1.54)	1.37 (1.26 to 1.49)	0.56
Measures of interaction				
Relative excess risk due to interaction	-0.06 (-0.21 to 0.08)	0.02 (-0.03 to 0.08)	0.01 (-0.04 to 0.06)	0.54
P for additive interaction	0.39	0.38	0.60	0.44
P for multiplicative interaction	0.33	0.98	0.70	0.66
Attributable proportion, %				
Lower birth weight	23.8% (-8.9 to 56.5%)	24.0% (6.0 to 53.0%)	24.0% (8.0 to 49.0%)	0.98
Unhealthy lifestyles	92.9% (61.7 to 124.1%)	70.0% (59.0 to 81.0%)	72.0% (62.0 to 83.0%)	0.38
Additive interaction	-16.7% (-48.6 to 15.3%)	6.0% (-10.0 to 23.0%)	1.0% (-13.0 to 16.0%)	0.45

*Cox proportional hazards models were adjusted for age, ethnicity (white, yes/no), family history of CVD (yes/no), as well as time-varying marital status (yes/no), living status (alone or not), menopausal status [premenopausal or postmenopausal (never, past, or current menopausal hormone use), women only]. †Unhealthy lifestyles include currently smoking, exercising <30 min/d at moderate intensity, DASH diet score in the bottom three fifths, body mass index ≥25 kg/m^2, and alcohol consumption >15 g alcohol/d in women and >30 g alcohol/d in men. ‡Test for between-study heterogeneity; for each lifestyle factor, each participant received a score of 1 if they met the criterion for high risk which were summarized to calculate the overall unhealthy score. Abbreviations: CHD=coronary heart disease; CI = confidence interval.
